# Holocene environmental change in Rotsee and its impact on sedimentary carbon storage

**DOI:** 10.1007/s10933-025-00361-3

**Published:** 2025-06-11

**Authors:** Cindy De Jonge, Nathalie Dubois, S. Nemiah Ladd, Longhui Deng, Niroshan Gajendra, Negar Haghipour, Carsten J. Schubert, Mark Lever

**Affiliations:** 1 Geological Institute, ETH Zurich, Sonneggstrasse 5, 8092 Zurich, Switzerland; 2 Department of Surface Waters Research and Management, Eawag, Swiss Federal Institute of Aquatic Science and Technology, Uberlandstrasse 133, 8600 Dubendorf, Switzerland; 3 Department of Environmental Sciences, University of Basel, Bernoullistrasse 30/32, 4056 Basel, Switzerland; 4 Institute of Biogeochemistry and Pollutant Dynamics, ETH Zurich, Universitaetstrasse 16, 8092 Zurich, Switzerland; 5 Institute for Energy Technology (IFE), Instituttveien 18, 2007 Kjeller, Norway; 6 Laboratory of Ion Beam Physics, ETH Zurich, Otto-Stern-Weg 5, 8093 Zurich, Switzerland; 7 Department of Surface Waters Research and Management, Eawag, Swiss Federal Institute of Aquatic Science and Technology, Seestrasse 79, 6047 Kastanienbaum, Switzerland; 8 Marine Science Institute, University of Texas at Austin, 750 Channel View Drive, Port Aransas, TX 78373 USA

**Keywords:** Lake system evolution, Sedimentary carbon, Ancient DNA, Py-GC/MS, Provenance

## Abstract

To assess the long-term impact of climate change and human influence on lakes and their sedimentary carbon storage, paleo-environmental approaches using well-dated lake sediment cores can be employed. Here, we reconstruct carbon mass accumulation rates for organic and inorganic carbon since 13 ka BP in Rotsee, a perialpine lake near the Swiss Alps, using a 12-m sediment core. A multiproxy approach (XRF, carbon and nitrogen isotopes, organic macromolecule chemical compositions, aDNA) was used to explore changes in the lake system that affect sedimentary carbon storage. The Early Holocene (11.8–7 cal ka BP) was characterized by a mixed phytoplankton and watershed-derived provenance of organic matter, and the deposition of inorganic and organic sedimentary carbon. Warming during the Holocene Thermal Maximum (9.8–8.8 cal ka BP) increased sedimentary carbon storage. In the Mid- to Late Holocene (7–1 cal ka BP), the sedimentary record indicates an increased influx of allochthonous, vascular plant-derived organic matter, and low production or conservation of phytoplankton-derived carbon. Organic carbon storage increased, while inorganic carbon became negligible. Larger deforestation events, potentially during Neolithic times (around 4 ka BP), but especially during Roman times (2 ka BP), coincided with further increased organic carbon MARs. Recent sediments, influenced by eutrophication in the last century, show higher carbon accumulation rates compared to earlier Holocene periods. Rotsee serves as a case study of how climate warming and human land use changes have influenced lake development and sedimentary carbon6 storage, with broader implications for understanding carbon dynamics in high altitude lakes and their future carbon balance.

**Supplementary Information:**

The online version contains supplementary material available at 10.1007/s10933-025-00361-3.

## Introduction

Present-day climatic changes impact highly sensitive alpine and subalpine lake ecosystems, their sediment composition and carbon storage potential (Moser et al. 2019). With increasing temperatures and glacier retreat, increased growth of vegetation and soil development (reviewed in Trautmann et al. 2023), and enhanced transfer of nutrients by leaching or deposition, may cause increases in lake trophic state (Pastorino et al. 2024). This climate-related eutrophication, as well as anthropogenically-driven eutrophication, have already increased storage of organic carbon in lake sediments (Anderson et al. 2014; Fiskal et al. 2019). Still, as average temperatures continue to increase impacting both the production and respiration of carbon (Gudasz et al. 2010), it remains difficult to predict whether lake sediments will be a future source or sink of carbon.

In the last 14 ka, the Alps and their foreland have experienced climate variability. This started with the warming following the last glacial maximum (22.2 ± 1.0 ka, Reber et al. 2014), which caused extensive foreland glaciers to recede. The variability in temperature and associated ecological impacts of the Bolling-Allerød interstadial (14.68–12.86 ka) and colder Younger Dryas stadial (12.86–11.61 ka) are well-recorded in lake sediments (e.g. Moscariello et al. 1998; Lotter et al. 2000; Samartin et al. 2012; Höhn et al. 2022). The Younger Dryas was followed by the Holocene (11.61 ka to present), a period of relative climate stability. Vegetation reconstructions and tree line changes suggest that the early and mid-Holocene were relatively warm (Wick and Tinner 1997; Nicolussi et al. 2005). This period, referred to as the Holocene Thermal Maximum (HTM), has been recognized in several studies (e.g. Renssen et al. 2012). The exact timing of the HTM is debated but studies propose it occurred from 7.5 to 5.9 ka in Austria (Vollweiler et al. 2006), or from 9.8 to 6.4 ka, with an intermittent cold episode from 8.8 to 7.8 ka, in Switzerland (Affolter et al. 2019).

These past climatic changes left an imprint on lacustrine sediments. For instance, warming, resulting in lake level drop, has been identified as a cause of eutrophication in Lake Lobsigen in the early Holocene (Ammann 1986). Climate changes, moreover, impact lake sediments by promoting the development of shoreline peat or vegetation (Ammann 1986) and/or lake infilling (Heiri and Lotter 2003). While this is a natural stage in the succession of a freshwater system, enhanced infilling and eutrophication can also be the consequence of human activities, such as increased sewage input (e.g. Hasler 1947; Fiskal et al. 2019), or increased outwash of nutrients due to soil erosion following deforestation (e.g. Haas et al. 2019). In the Swiss alpine realm reconstructed vegetation dynamics indicate that anthropogenic deforestation started having a substantial impact around 5 ka BP (Wick and Tinner 1997; Nicolussi et al. 2005), and subsequently increased carbon storage in lake sediments (Kastowski et al. 2011).

Rotsee is a small, monomictic lake (0.48 km^2^) in the Swiss alpine foreland that was formed after the retreat of the Reuss glacier (14.6 ka), as suggested by the age of a terrestrial macrofossil (C823, 14570 ± 240 cal ^14^C age) from a shoreline peat core (Lotter 1988). Past sedimentological and paleo-ecological descriptions of Rotsee sediments, based on pollen, plant macrofossils, diatom frustules, stable isotopes (Lotter 1988; 1989) and oxygen isotopes (Verbruggen et al. 2010; Ursenbacher et al. 2020), show that Rotsee sediments recorded a well-expressed Late-Glacial interstadial, Younger Dryas, and Holocene. At the Younger Dryas/Holocene transition, an up to 4 °C warming of the surface water temperature during the summer has been reconstructed (Verbruggen et al. 2010). The age boundaries of the HTM at Rotsee (pollen zones VI and VII in Lotter 1988) agree with previously determined boundaries (8–5 ka BP; Welten 1982). Lotter (1988) describes that Rotsee was originally deep and oligotrophic, and rapidly became meso- to eutrophic as the soils in the surrounding watershed developed. This increase in trophic state coincided with the establishment of extensive shoreline Alder Carr vegetation starting at 7 cal ka BP (Lotter 1988). Despite the oligotrophic conditions, chironomid relative abundances show that during the Younger Dryas and earliest Holocene, the hypolimnion had decreased oxygen concentrations (Ursenbacher et al. 2020), a condition that was also reported for the last 2 ka (Züllig and Rheineck 1985) following deforestation of the watershed by Roman settlers (Lotter 1988). More recently, as a result of human population growth that increased the input of untreated wastewater after 1850 (Stadelmann 1980), Rotsee became highly eutrophic. This resulted in the formation of a seasonal chemocline where anoxia extended to the photic zone, as indicated by the occurrence of okenone, a biomarker for anoxygenic phototrophic purple sulfur bacteria, in sediments deposited since 1850 (Züllig and Rheineck 1985).

Because of its long record of climate, vegetation, and anthropogenic changes, Rotsee sediments provide an excellent opportunity to investigate how these changes affect the long-term burial and preservation of sedimentary carbon pools. Based on a multiproxy approach we here test the hypothesis that climate warming from the Late Glacial to the early Holocene contributed to an increase in trophic state, primary productivity, and associated organic carbon burial rates in Rotsee. We furthermore postulate that this increase was accelerated early on by warming during the HTM, and later by trophic state alterations due to anthropogenic land-use changes, with the latter ultimately more significantly affecting carbon storage than natural climatic shifts.

## Methods

### Core collection and on-site subsampling

Three short cores and two long cores were collected at a water depth of 5.5 m (47°04ʹ27.81ʺN, 8°19 ʹ 25.7ʺE WGS 84; 667230/214087 LV95) between 03/10/2021 and 05/10/2021. The short cores (40–60 cm long) were collected from a vessel using a gravity corer with clear plastic liners (UWITEC; inner liner diameter 90 mm). The two long cores (sections ROT21-1-1 to ROT21-1-5 and ROT21-1-6 to ROT21-1-9) were taken 4 m apart from each other, using a shoreline moored platform using a piston coring system with a manual hammer, without a re-entry cone (UWITEC; inner liner diameter: 59.5 mm). Long cores were taken in sections of 3 m (except for 1 section that was 2 m). The recovery of two parallel long cores, vertically offset by one meter, was necessary to obtain a high-quality, complete sedimentary sequence. All short and long cores were brought onshore for sampling immediately after core recovery. Short cores were maintained in vertical position and sampled by extrusion, whereas long cores were first accessed horizontally through ‘windows’ that were cut into the core liner. From each depth sampled, sediments for determination of porosity and bulk density, DNA analyses and macromolecular organic matter analyses were collected with sterile cut-off syringes. Samples for DNA extraction were immediately frozen in liquid N_2_, before storage at – 80 °C, whereas samples for organic matter analysis were frozen and stored at – 20 °C. Afterwards, the core sections were split lengthwise before subsampling 2 cm slots for bulk carbon and nitrogen analyses using solvent cleaned spatulas. One core half (the so-called archive half) remained intact for imaging and XRF scanning.

### XRF scanning

Elemental compositions were measured at 5 mm resolution using a µXRF core scanner (Avaatech XRF) with an Oxford 100 W X-Ray tube and a rhodium anode equipped with a Canberra X—PIPS/DSA 1000 (MCA) detector. Prior to analysis, core surfaces were flattened to ensure uniform scanning and covered with 4 µm Ultralene foil. Elemental groups with lower energy levels were measured at 10 kV (1500 A, no filter, 15 s exposure), while mid-energy groups were measured at 30 kV (2000 A, Pd thin filter, 40 s exposure). Prior to determining the variability in XRF parameters (excluding Mo, Ar and coherent and incoherent scatter) using a principal component analysis (Supp. Fig. 1), the cps counts were transformed using a centered log-ratio transformation (Bertrand et al. 2024) and scaled. Based on untransformed cps counts (Supp. Fig. 2), selected XRF log-ratios were calculated.

### Dating and age model

**Fig.1:**
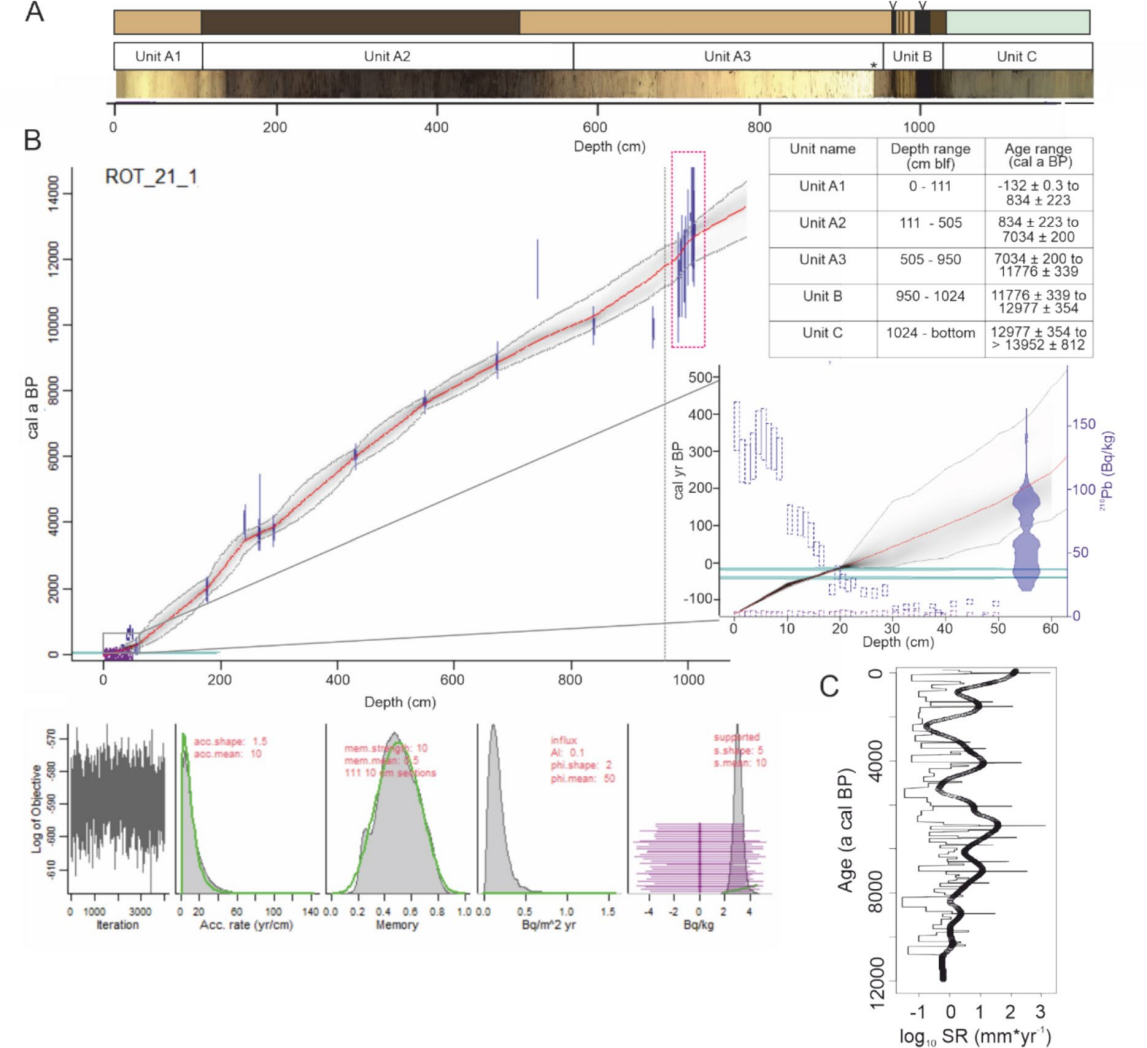
Visual overview of the Lake Rotsee core with (**A**) a photographic image of the sedimentary units, with an asterisk indicating a change in picture exposure impacting image lightness within Unit A3. A simplified lithological log is plotted, with layers of macrophyte material indicated (v). The proposed age model (**B**) plotted is based on 19 ^14^C dated macrofossils and unsupported ^210^Pb measurements (inset). The depth range of ^14^C ages that is based on aquatic macrophytes is indicated in a red rectangle, and depth and age intervals of core sections are tabulated. Also inset are the summary of rplum model parameters. Panel C reports the estimated sedimentation rates (log_10_ mm * yr^−1^), where the sedimentation rate at a cm resolution and the smoothed trend are depicted

The top 50 cm of a short core was sectioned at 1 cm resolution and used for ^210^Pb and ^137^Cs dating (Fig. 1A; Supp. Table 1A). ^137^Cs peaks were determined to date the sediment layers deposited in 1987 and 1963 Anno Domini (AD). In addition, radiocarbon dating on 19 macrofossils, including 12 seeds, leaf remains and twigs of terrestrial plants, and 7 macrodetrital remains of aquatic macrophytes was performed (Fig. 1B, Supp. Table 1B). After wet sieving, macrofossils were subjected to an acid-alkali-acid treatment at room temperature (Norris et al. 2020) to remove carbonates, acid soluble humic material, and humic acids. At two depths, bulk sediments were acidified using fumigation (described in Haas et al. 2019) and weighed in for ^14^C dating, with the aim of constraining the reservoir age during the Younger Dryas (Supp. Table 1C). The reservoir age was used to correct the uncalibrated ^14^C ages measured on the aquatic macrophytes. ^14^C measurements were carried out on an Accelerator Mass Spectrometer (AMS) with an Elemental Analyzer unit (EA) using the MIni CArbon DAting System (MICADAS) at the Laboratory for Ion Beam Physics of the Eidgenössische Technische Hochschule (ETH) in Zurich. An age-depth model was created using rplum, which allows unsupported ^210^Pb values, ^137^Cs ages and uncalibrated ^14^C ages to be combined based on Bayesian statistics (Blaauw and Christen 2011). Radon measurements are available as estimates of supported ^210^Pb, assumed constant by the model. A memory strength of 10 and memory mean of 0.5 is used. In this model, ^14^C ages are converted to calendar ages using the INtCal20 calibration curve (Reimer et al. 2020).

### Bulk nitrogen and carbon content and isotopes

Sediments were freeze-dried and homogenized using an agate mortar and pestle. Total nitrogen (%; TN) and δ^15^N-TN values were determined on between 3 and 200 mg of unacidified sediments using an EA-IRMS system composed of a Vario Pyro Cube coupled to a Isoprime 100 IRMS (Elementar, Germany). Repeated measurement of reference materials Acetanilide #1 (Schimmelmann, USA, δ^15^N = +1.18 ± 0.02) and High Organic Sediment Standard (HEKAtech, Germany, δ^15^N = +4.32 ± 0.20) were used to determine the instrument precision, which was determined to be generally below 0.05 ‰ δ^15^N for the Acetanilide standard, and below 0.07 ‰ δ^15^N for the sediment standard. Offsets between the measured and known δ^15^N values of the Acetanilide standard (average offset = 0.19 ± 0.08) were used to correct the δ^15^N-TN values of the bulk sediments. The contents of total carbon (TC), total organic (TOC) and total inorganic (TIC) carbon were measured on 50 mg of sample weighed into a ceramic crucible, on the SoliTOC® Cube (Elementar Analysensysteme, Germany). The reported TOC is the summed amounts of TOC400 and refractory organic carbon (ROC), with TOC400 determined at 400 °C and ROC between 400 and 600 °C, and TIC between 600 and 900 °C. Low (B2152) and high organic carbon content standards (B2150) from Elemental Microanalysis (United Kingdom) were run with each batch to determine the instrument accuracy, which was determined to be 98.9 ± 0.6%. δ^13^C-TOC of bulk sediments was measured on an EA-IRMS system, EA Vario Pyro Cube (Elementar Analysensysteme, Germany) and Isoprime IRMS (GV Instruments, UK), after acidification. For acidification, samples with inorganic carbon were subjected to a 1N HCl treatment until no more visible reaction occurred. To calibrate the instrument an Acetanilide #1 (Schimmelmann, USA, δ^13^C = -29.52 ± 0.01) standard was used, as well as a High Organic Content Sediment (SA990894; δ^13^C = -28.85 ± 0.10) and Low Organic Soil (SA33802152; δ^13^C = − 22.88 ± 0.10) standards from Hekatech Analytics. In general, instrument precision during the runs was below 0.06 ‰ δ^13^C for Acetanilide and below 0.16 ‰ δ^13^C for the sediment and soil standard, and an accuracy better than 0.02 for δ^13^C for Acetanilide and 0.1 ‰ δ^13^C for the sediment and soil standard. No corrections of the δ^13^C values were performed.

### Bulk compound classes and hydrocarbons

To determine the OM macro-molecular composition, pyrolysis gas chromatography mass spectrometry was used, following the set-up as described in Gajendra et al. (2023). Between 100 - 500 μg of freeze-dried sediments were weighed into autosampler containers (Eco-cup SF, Frontier Laboratories, Japan) and pyrolyzed at 450 °C and 650 °C, according to Tolu et al. (2015). The pyrolizer was connected to an autosampler (PY-2020iD and AS-1020E, FrontierLabs, Japan) and to a GC/MS system (Trace 1310, Thermo Scientific and ISQ 7000, Thermo Scientific) equipped with a DB-5MS capillary column (30 m × 0.25 mm i.d., 0.25 mm film thickness; J&W, Agilent Technologies AB, Sweden). Chromatograms were analyzed in R (version 2.15.2, 64 bits) based on a pipeline that identifies common mass fragments as pyrolysis products (Tolu et al. 2015). Pyrolysis products were then classified and annotated according to Tolu et al. (2015) and Ninnes et al. (2017). On average 27% of the total peak area occurred in peaks that didn’t provide conclusive structural information. Areas of individual compounds within each compound class were summed up (Supp. Table 2 for identity of individual compounds), and compound classes expressed as relative abundances (peak area of each compound class relative to total characterizable Py-GC/MS peak area). To summarize the main trends in compositional variability, a PCA was performed based on the standardized fractional abundance of the compound classes (Supp. Fig. 3).

### Mass accumulation rates

Dry bulk density values, the mass (weight) of the dry solids divided by the total volume of the wet sample, were measured on 7 mL of sediments sampled with a cut-off syringe, based on weights before and after drying (*n* = 68). Because of the presence, and post-sampling expansion, of free methane gas in the sediments, the measured DBD values should be interpreted as a potential under-estimation. Using the linterp command from the astrochron package (Meyers 2014), the bulk dry density values were interpolated at a 1 cm resolution. Mass accumulation rates (MAR) were then calculated by multiplying the interpolated dry bulk density with measured weight percentages of TOC, TIC, and normalized per year, using a smoothed sedimentation rate (autoplot, smoothing with a smoothing width of 800, using the astrochron package; Meyers 2014). Supp. Fig. 4 shows the variability of measured and interpolated parameters that are used to calculate the MAR values through time.

### aDNA analysis

Sedimentary DNA was extracted according to Lysis Protocol I of Lever et al. 2015. Briefly, 0.2 g of sediment was added to 2-mL screw-cap tubes filled to 15 % with 0.1 mm Zr beads. For the vast majority of samples, 100 µl of 10 mM adenosine triphosphate (ATP; prepared by dissolving adenosine 5’-triphosphate disodium trihydrate in molecular grade water) solution was added to reduce DNA sorption. The only exceptions were deep glacial clay layers, in which recovery was significantly enhanced by increasing the ATP concentration to 300 mM. 0.5 ml of lysis solution I was added to all samples (Lever et al. 2015). Samples were then shaken for one hour at 50 °C (600 rpm; ThermoShaker, Eppendorf), and subsequently washed twice with cold 24:1 chloroform-isoamyl alcohol and precipitated with linear polyacrylamide (20 µg ml^-1^), 5 M sodium chloride and ethanol. The pellets were dried using a vacuum centrifuge (50 °C; Thermo Fisher Scientific, USA), resuspended in molecular grade water and purified with the CleanAll DNA/RNA Clean-up and Concentration Micro Kit (Norgen Biotek Corp., Canada; Protocol A). All extracts of samples and extraction negative controls (extraction blanks) were stored at – 80 °C. Eukaryotic 18S rRNA and rbcL genes were quantified by real-time PCR (Lightcycler® 480; Roche) with SYBR®Green as dye. Eukaryotic 18S rRNA genes were amplified using the All18S primer pair (Deng et al. 2020), whereas chloroplast genes encoding the large subunit of ribulose-1,5bisphosphate carboxylase (rbcL) were quantified using published assays for vascular plants (Willerslev et al. 2003), Chlorophyta and Ochrophyta (both Han et al. 2022).

## Results

### Core description and age model

The first (core section ROT21-1-1 to ROT21-1-4) and second borehole (core sections ROT21-1-5 to ROT21-1-8) were aligned based on 25 tie points of the XRF traces Zr, Co, Ca, P/Ti and Ca/Ti (see data repository). The alignment allowed 5–20 cm sediment sections at the top of each 3 m section to be identified as infill; these sediment layers were removed from further analysis. Based on the compiled core ROT21-1, the sedimentary record of Rotsee has 3 lithological units (Fig. 1A), that reflect visual changes in the sediment color. The deepest section (Unit C) consists of a homogenous deposit of 2.8 m composed of clays. This is overlaid by a narrow section (70 cm; Unit B) of banded sediments, composed of light-colored clays, interspersed with dark brown layers of well-preserved macrophyte material and brown organic-rich sediments (gyttja). Unit A represents a period of continuous sedimentation, composed of 4.5 m of non-varved calcareous gyttja (Unit A3), gradually changing into 4 m of non-varved non-calcareous gyttja (Unit A2), with the surface 1 m showing a gradual return to calcareous gyttja (Unit A1). The general core description matches the composition of core RL-240 and RL-250 taken in the year 1985, described by Lotter (1988), with some notable differences. For instance, the Laacher See Tephra was described in core LR-250 at 776 cm depth below lake floor (blf) but while we would expect it towards the bottom of Unit B, it was not recognized at our site. On the other hand, layers of macrophyte material (Unit B) are present in our core, that were not encountered in the older cores.

The Bayesian age model (Fig. 1B) shows that the top 1024 cm (Unit A–B) cover the last 13 cal ka BP (± 0.35 ka), with ‘present’ defined as the year 1950. Within Unit B several reversals of non-calibrated ^14^C ages are documented, on the order of 70–400 years. However, these age offsets do not exceed the summed error of the estimated ages, which is composed of the measurement error and the error inherited from the reservoir age correction (after Soulet 2015; Supp. Table 1C), and are therefore not interpreted as age reversals. Within Unit A, 9 of the 12 dated plant macrofossils overlap with the 95% confidence interval of the age-depth model. The top of the composite profile is determined by the alignment of the short core XRF Pb profile. Based on a Bayesian model of ^210^Pb activities, where unsupported ^210^Pb was calculated by subtracting ^226^Ra activity from total ^210^Pb, an age of 0 yr is modeled at 8.5 cm depth. While the radio-isotopic profile for ^210^Pb generally follows the expected exponential ^210^Pb decay, sediments deposited between 2 and 5 cm depth have a decreased ^210^Pb activity (Fig. 1B). A decrease of ^210^Pb activity in recent sediments has been described before (Baud et al. 2023) and has been partially explained by eutrophication and/or acidification of the lake. Since the sediment ages based on the ^210^Pb model and two identified ^137^Cs peaks (Supp. Table 1) match, sediments above 8.5 cm depth are interpreted to reflect modern material. Smoothed sedimentation rates (Fig. 1C) are on average 2.3 mm yr^−1^, with notable increases to ~ 2.5 mm yr^−1^ around 9, 4.2 and 1.6 ka BP, increases beyond 4.5 mm yr^−1^ around 7 ka BP, and the highest values, above 5 mm yr^−1^ in sediments deposited in the last 0.5 ka.

Based on the timing of previously published climate reversals at Rotsee, Unit C is thus contemporary with the deposition of clays during the last Glacial Interstadial, Unit B was deposited during the Younger Dryas (12,976 ± 354 to 11,775 ± 340 cal a BP), and Unit A covers the Holocene, with Unit A2 deposited during the Mid- to Late Holocene (7034 ± 199 to 834 ± 221 cal a BP).

### Sedimentary composition with depth

**Fig. 2:**
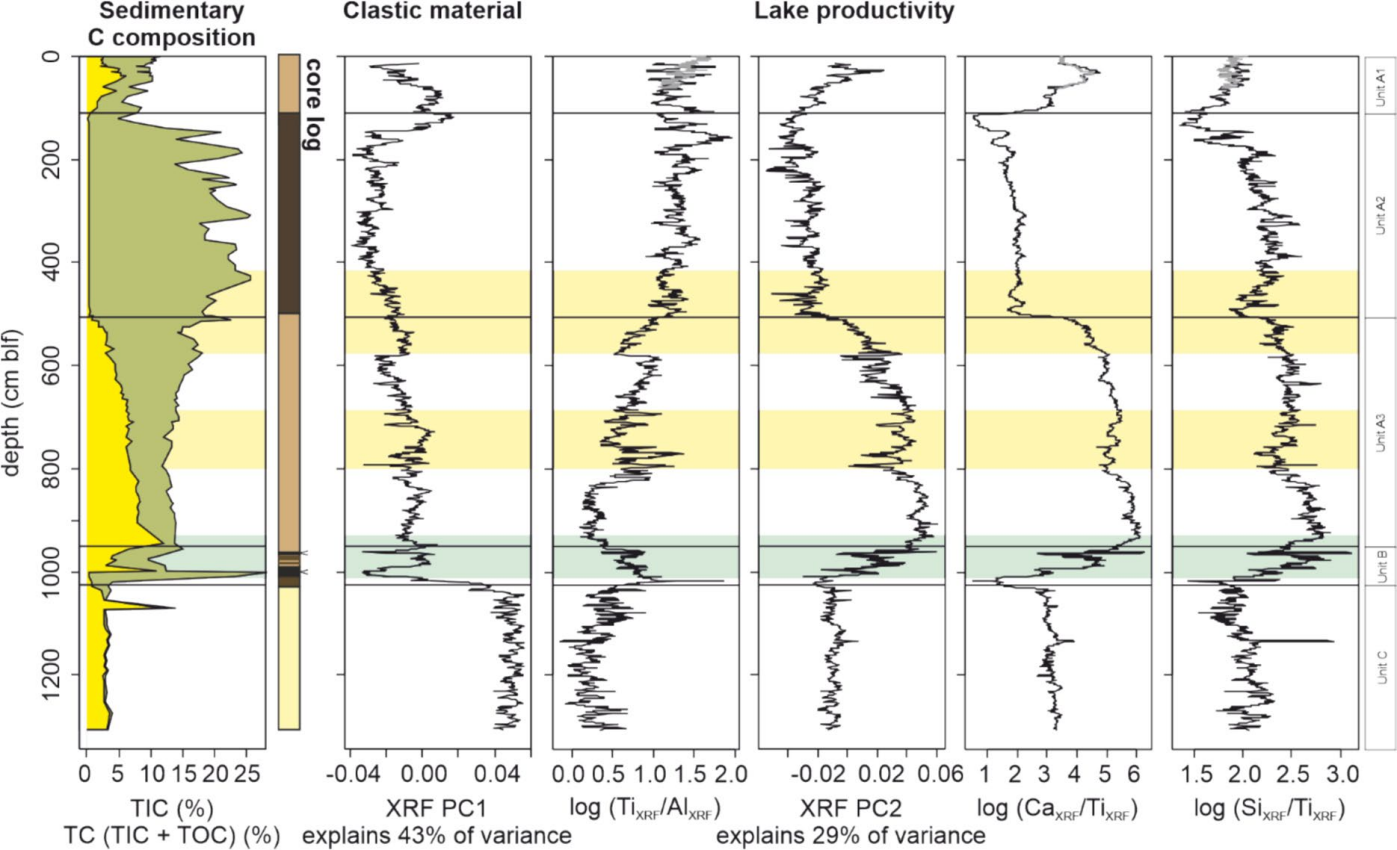
Overview of main Rotsee parameters plotted against composite depth (cm below lake floor (blf)); sedimentary content (%) of total inorganic carbon (TIC) and total carbon (TIC + total organic carbon (TOC)), the simplified lithological log (core log), the values of the first two principal components of the XRF variability (XRF PC1, XRF PC2) with percentage of variance explained indicated and XRF based log-ratios Ti/Al, Ca/Ti, Si/Ti. Parameters are plotted grouped per sedimentary parameter (Sedimentary carbon (C) composition, clastic material and lake productivity)

Total inorganic carbon (TIC) and total organic carbon (TOC) show a large downcore variation in dry weight percent (1.6–28 % for TOC, 1.8–12 % for TIC; Fig. 2). In Unit C, Total Carbon (TC) is represented almost entirely by inorganic carbon, which reaches up to 4 % (dry sediment weight). Unit B shows strong fluctuations in TOC and TIC, with TIC and TOC reaching 10 and 25 % respectively. In the transition to Unit A3, TIC increases and TOC decreases again, only to be followed by a gradual increase in TOC (from 5 % to 25 %) and decrease in TIC (from ~12 % to < 0.5 %) throughout A3. In Unit A2 sedimentary carbon is represented almost exclusively by organic carbon, with values of 15–25 % except for a decrease to 5 % in the transition to A1. In A1, TOC remains at the current values of 5 %, and TIC increases to values of 2–5%.

To further investigate changes in the sedimentary composition with depth, the elemental composition was characterized by XRF scanning. Different sources of variability are reflected by the scores on the principal component axes (Supp. Fig. 1). PC1 is mainly driven by variations in the contents of terrigenous (Si, Al, K, Mg, Ti) elements (Supp. Fig. 1) that indicate varying contributions of fine-grained clastic material. The downcore variation in PC1 values (Fig. 2) is thus interpreted as high clastic material in Unit C, low inputs throughout Units B, A3, and most of A2, and increased again in the top sediments of Unit A2 (140 cm) and A1. Complementary analyses of Ti/Al ratios are generally interpreted as a proxy for grain size, with Ti reflecting larger grain sizes (i.e. Zhao et al. 2011). Sedimentary increases in Ti/Al log-ratio values are generally interpreted to respond to an increase in grain sizes following land use changes (soil erosion, i.e. Olsen et al. 2010), or an increase in terrigenous riverine input (Lim et al. 2019). At Rotsee, low values are encountered in the glacial clay layer in Unit C (Fig. 2). Throughout Units B and A, Ti/Al log-ratio values increase gradually, reaching maximum values towards the top of A2. Notably, there is a negative correlation between log-ratio Ti/Al values and the amount of clastic material as captured by PC1 values (r = − 0.72, p < 0.01).

The second principal component (PC2) is driven by variations in Ca, Sr and P (Supp. Fig. 1). Downcore PC2 values generally follow the TIC content (Fig. 2). PC2 is interpreted to reflect variations in the sedimentary calcium carbonate (calcite) content, with Sr being a common trace element in calcite. Matching the inorganic carbon values, PC2 values are intermediate in glacial clays (Unit C), and mostly increase throughout Unit B, reaching their highest values at the base of A3, before they decrease to low values (A2) and increase again in the most recent sediments (Unit A1). The orthogonal variation to PC1 indicate that PC2 is not driven by clastic material. PC2 and Ti/Al log-ratio values, show an inverse correlation (r = − 0.57, p < 0.001). Instead, PC2 correlates strongly with the log-ratios of Ca/Ti (r = 0.95, p < 0.001) and Si/Ti (r = 0.75, p < 0.001). The good correlation of both log-ratios indicates that they capture biogenic calcite and silica, respectively, and can be used to reconstruct changes in primary productivity, as shown previously by Liu et al. (2013). Here, biogenic calcite is interpreted to be dominantly calcite minerals that form from supersaturated lake water through the impact of phytoplankton blooms on the carbonate equilibrium of the water (Mueller et al. 2026). Detrital inputs of Ca-bearing minerals are generally of minor importance, and at least in recent periods, the carbonate content of Rotsee was a good proxy for primary productivity (Lotter 1989). If K/Ti is interpreted as a proxy for fine-grained minerogenic supply to the lake, Supp. Fig. 5 shows the large changes in response size between Ca/Ti, whose precipitation is biologically induced, and the background supply of minerogenic sediments (K/Ti). The bulk of Si delivered to lakes is bio-available, and diatoms precipitate the bulk of dissolved Si in Swiss lakes (Lake Lugano; Hofmann et al. 2002). As diatom valve counts of up to 10 * 10^6^ valves per g dry weight have been recorded in Rotsee (Lotter 1988), it is likely that the bulk of the silica as estimated by the Si/Ti log-ratio is biogenic silica.

XRF log-ratios of redox-sensitive elements Mn, Fe and S (Mn/Ti, Fe/Ti, S/Ti log-ratios) are evaluated to outline their proxy potential for redox conditions (Supp. Fig. 5). The Mn/Ti and S/Ti logratio values both increase from Unit C to Unit A, and all three ratios correlate with each other within Unit A (0.59 < r < 0.95, p < 0.01). While Mn/Ti and Fe/Ti generally show an increase in Unit A2, S/Ti does not increase in these sediments. Furthermore, peaks in Fe/Ti logratio values occur between 260 and 300 and at 344–354 cm; Supp. Fig. 5) that are mimicked by increases in the P/Ti log-ratio value, but not by the Mn/Ti and S/Ti log-ratios. While the redox-sensitive proxies discussed here thus all reflect sedimentary anoxic conditions in Unit A and B, compared with Unit C, variation in individual ratios within Unit A indicates that additional environmental variability impact the sediment content of Mn and Fe.

### Carbon accumulation rate and provenance through time

**Fig. 3:**
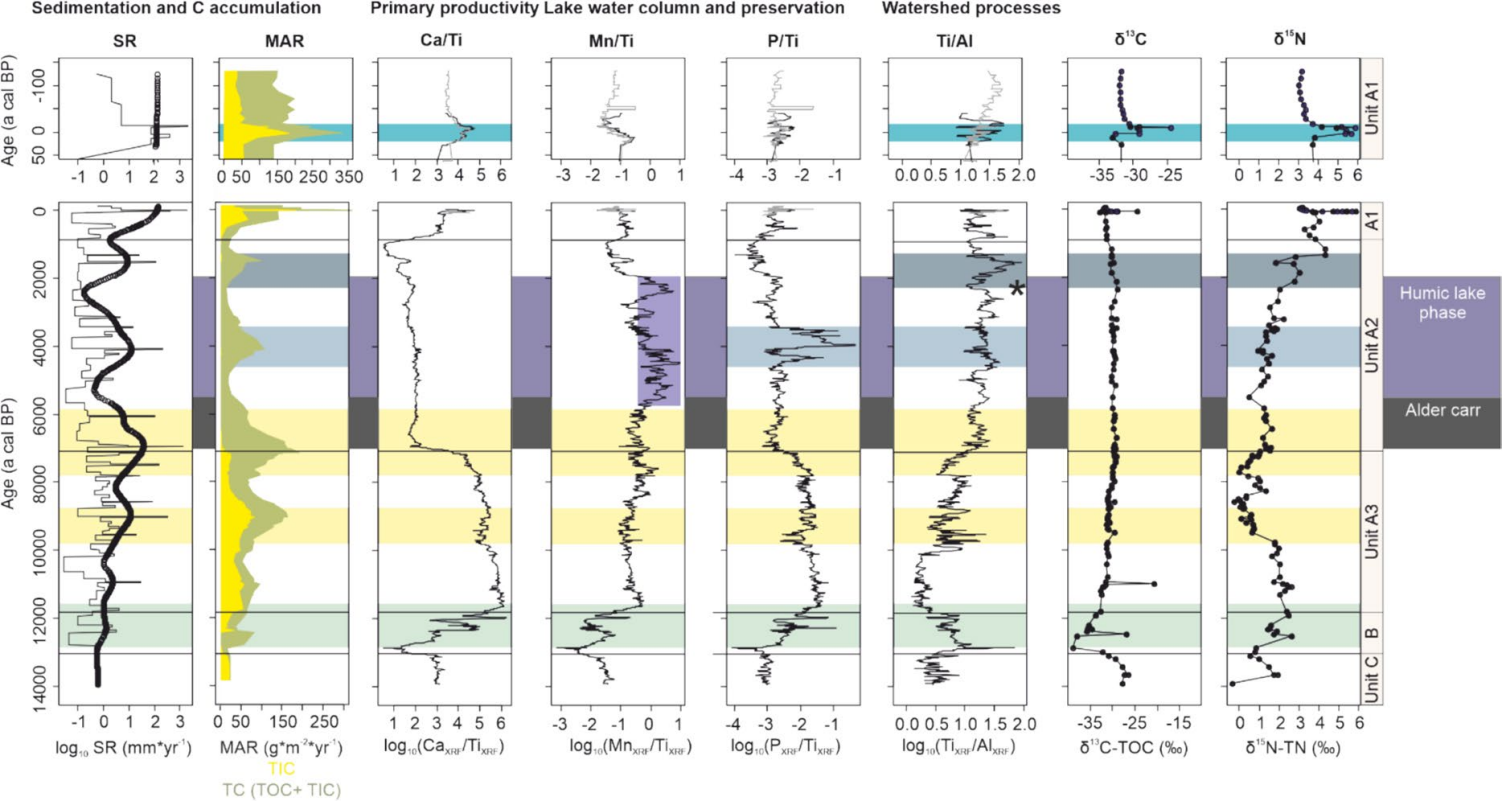
Sedimentation rate (SR, log_10_ mm*yr^−1^), mass accumulation rates (MAR) of sedimentary total inorganic carbon (TIC) and total carbon (TIC + total organic carbon (TOC)), Ca/Ti log-ratio, Mn/Ti log-ratio, P/Ti log-ratio, Ti/Al log-ratio, bulk δ^13^C-TOC, and bulk δ^15^N-TN values are plotted against time. The presence of an extensive Alder carr shoreline forest (Lotter 1988) and the proposed time extent of a humic lake phase (this study) are plotted as background panels. Younger Dryas is indicated as green background panels, Holocene Thermal Maxima are indicated in yellow. Periods of increased MAR that are not linked to these climate zones, are indicated in shades of blue, with parameters included in the discussion showing the same shading. The most recent 100 years are replotted on a more detailed scale

The large changes in TIC and TOC content result in variable individual mass accumulation rates (MARs) of organic and inorganic carbon through time (Fig. 3). The inorganic carbon MAR becomes substantial only in early Holocene sediments with a sustained maximum of 50 g*m^-2^*yr^-1^ between 11.9 and 8.5 ± 0.29 cal ka BP in Unit A3. Afterwards it decreases, with no accumulation of inorganic carbon in Unit A2. Intermediary inorganic carbon MAR values are observed in Unit A1, with a pronounced maximum around 1950 AD (Fig. 3). Organic carbon MARs are intermediate in Unit A3 and A1, reaching maximum values (170 g*m^-2^*yr^-1^) at the boundary between Unit A3 and A2 (between 7.4 and 6.5 ± 0.2 cal ka BP). Within Unit A3 a maximum of 80 g*m^-2^*yr^-1^ is reached between 9.1 and 8.7 ± 0.19 cal ka BP. Within Unit A2 a maximum of 90 g*m^-2^*yr^-1^ is observed between 4.3 and 3.6 ± 0.2 cal ka BP and between 1.6 and 1.3 ± 0.2 cal ka BP. The total carbon accumulation rate is highest in recent sediments (330 g*m^-2^*yr^-1^ at 1948 ± 10 cal a AD) followed by the total carbon accumulation rates at the transition from Unit A3 to Unit A2 (up to 190 g*m^-2^*yr^-1^, between 7.4 and 6.5 ± 0.2 cal ka BP; Fig. 3). The composition of bulk organic matter, described with TOC/TN and bulk δ^13^C-TOC values, together with the proposed ranges in these bulk parameters for bacteria, phytoplankton and C_3_ plants, already indicate long-term changes in the provenance of organic matter in the different Units (Supp. Fig. 6). Bulk δ^13^C-TOC values show low values in Unit B, afterwards gradual increase to – 28 ‰, with generally low variability in Unit A (Fig. 3). Bulk δ^15^N-TN has a minimum (∼0 ‰) value in the upper half of Unit A3, showing a gradual increase to values of 2 ‰ in Unit A2 and of 5 ‰ in Unit A1. The increase is most pronounced in sediments shallower than 175 cm blf.

**Fig. 4:**
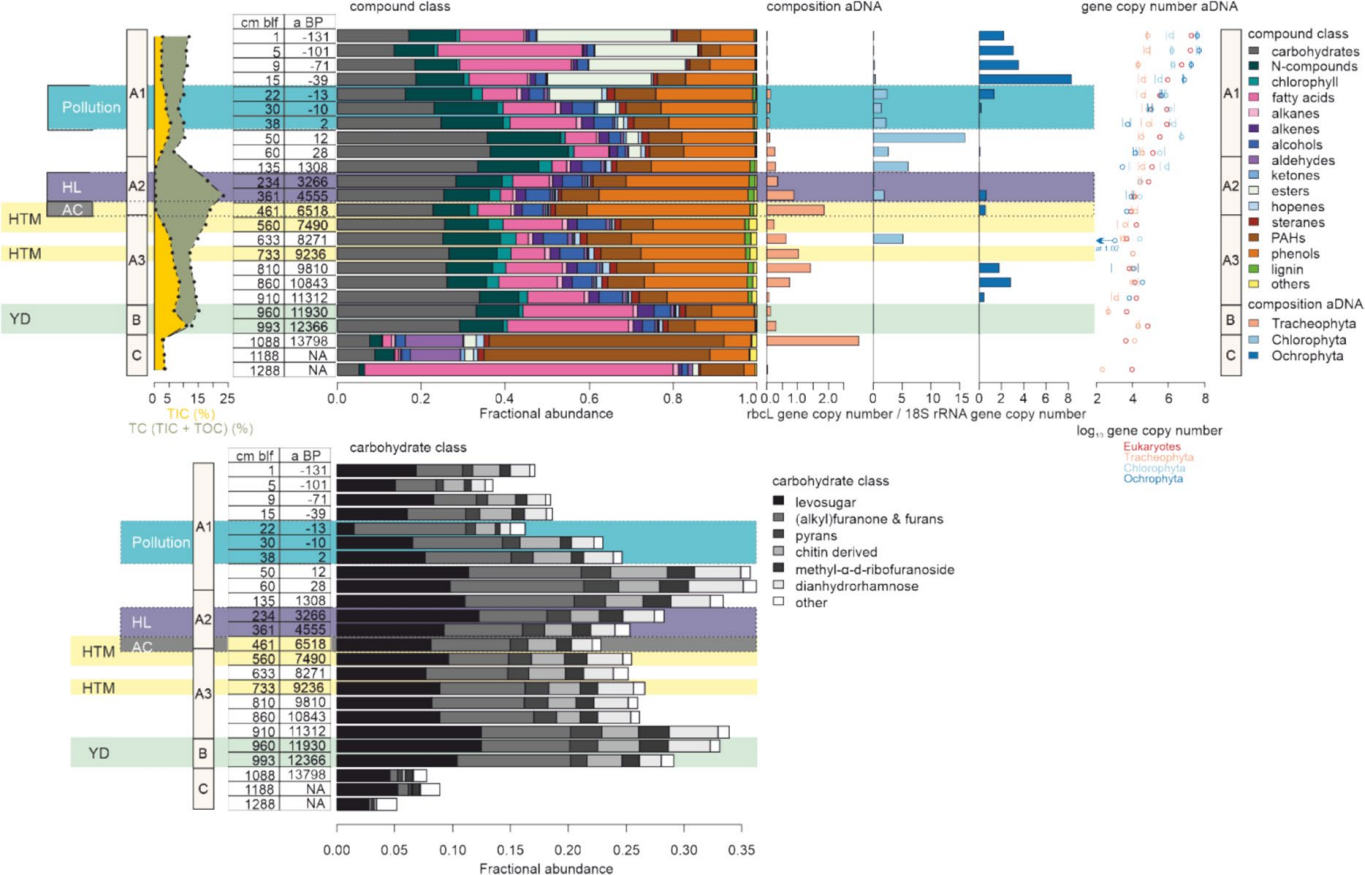
In comparison with the sedimentary content (%) of total inorganic carbon (TIC) and total carbon (TIC + total organic carbon) (for higher resolution, see Fig. 2), the distribution of organic compound classes in Rotsee sediments is plotted (Supp. Table 2 for composition of compound classes), with the carbohydrate composition replotted on a separate scale. The relative abundance of aDNA of Tracheophyta, Chlorophyta and Ochrophyta rbcL gene copies compared to the Eukaryotic 18S rRNA gene copies are plotted, as well as the gene copy number changes with depth. Background color of the tabulated ages and depths indicate Holocene climate zones, with Younger Dryas indicated in green and Holocene warm periods are indicated in yellow. The presence of an extensive Alder carr shoreline forest (AC: Lotter 1988) and the proposed time extent of a humic lake phase (HL: this study) and the extent in time of cultural eutrophication (Pollution, depth range reflects sediments with increased MARs [Fig. 3]) are plotted as background panels

To investigate the potential molecular drivers behind the observed variations in δ^13^C-TOC and δ^15^N-TN, we investigated the macromolecular composition of the sedimentary organic matter (Fig. 4). Clay sediments (Unit C) have a distinct macromolecular composition, characterized by either increased fatty acids or PAHs and aldehydes (13,798 ± 722 cal a BP and older). Lacustrine sediments overlying this section show an increase in carbohydrates that can be attributed to an increase in furanones, pyrans, chitin-derived sugars, methyl-α-d-ribofuranoside and dianhydrorhamnose (Fig. 4). In Unit A2 and A3, increased contributions of lignin (0.7–1.4%) and chlorophyll (2–4%) and increased contributions of phenols (22–39%) are observed. Phenols become especially abundant compounds (30–39%) in sediments deposited between 6600 and 3200 cal a BP (n = 3). In Unit A1, fatty acids represent 27–35% at 5–9 cm blf (modern sediments), and esters represent 13–32% of organic matter above 22 cm blf (60 ± 2 cal a BP; Fig. 4). N-compounds show a maximum (19%) at 60 cm blf (28 ± 97 cal a BP). A PCA analysis (Supp. Fig. 5) shows that the main variation in macromolecular composition is caused by the increase in the fractional abundance of esters and fatty acids in the surface sediments (PC1; Supp. Fig. 5). The second direction of variation concerns lignin, phenol and chlorophyll compounds that anti-correlate with N-containing compounds and carbohydrates, showing substantial variation across the whole core (PC2; Supp. Fig. 5).

To compare macromolecular organic matter compositions with the partial diversity of primary producers, Fig. 4 also shows the copy numbers of total eukaryotic 18S rRNA genes, and of rbcL genes belonging to Tracheophyta (vascular plants), Chlorophyta (green algae), and Ochrophyta (mainly diatoms). Gene copy numbers show a strong initial decrease with depth, with both 18S rRNA and rbcL gene copy numbers decreasing (100 to 10,000-fold for Eukaryotes and Ochrophyta respectively) in the most surficial sediment layers (0–10 cm). A subsurface maximum is present in sediments that were deposited in the last 50 years (12 ± 13 for Chlorophyta, 0 ± 10 for Eukaryotes and − 11 ± 8 for Tracheophyta, in cal a BP). After the initial decrease in gene copy numbers in the last 100 years, counts of eukaryotic DNA remain stable throughout the core. 18S rRNA genes of Eukaryotes and rbcL genes of Tracheophyta are found conserved in sediments of over 14 ka old (Unit C to Unit A1), while rbcL of Ochrophyta and Chlorophyta were found in sediments from over 11 ka and 8 ka old, respectively. In the early Holocene, the gene ratio of Tracheophyta rbcL to total 18S rRNA gene copies increases in pace with the compound groups of phenols, lignins and chlorophylls. Chlorophyta rbcL genes show a relative increase in the late Holocene, matching a period of increased N-compound and carbohydrate contributions. Ochrophyta rbcL genes show an increase in the most recent sediments, with a maximum at 15 cm blf (− 39 ± 1 cal a BP, 1989 AD).

## Discussion

### Natural successional and trophic changes in the young Rotsee system driven by climate

Climate warming from the Late Glacial to the middle Holocene caused marked changes in sedimentary carbon (Fig. 3) that indicate changes in lake trophic state. In the glacial clays, which are characterized by high inorganic and low organic carbon content, the TOC/TN ratio values and bulk δ^13^C-TOC values (Supp. Fig. 4), and high fatty acid, esters, and aldehyde contents indicate organic matter that is dominated by small amounts of bacterial biomass. The high contents of labile fatty acids, esters, and aldehydes indicates either a high preservation efficiency of microbial biomass that was deposited over 13 ka, or—perhaps more likely—present-day microbial populations that inhabit these glacial clays. This subsurface microbial community was also proposed for glacial clay layers underlying alpine Lake Cadagno (Berg et al. 2022; Gajendra et al. 2023). In contrast, the low contributions of lignin and phenolic compounds to the sediments (Fig. 4) imply minimal vegetation around Rotsee in this period (e.g. Bader 1957, Lotter 1988). PAHs are present and potentially derive from grassy vegetation fires. Glacial mass accumulation rates of carbon are not constrained, but were presumably low, based on potentially high mass accumulation rates and low TOC and TIC content.

During the Younger Dryas, the presence of distinct layers of macrophytes, identified as *Characeae* sp., coeval with a decrease in the supply of glacial clays (Fig. 2), attest to clear water conditions with high light availability at the lake floor. The well-preserved layers of macrophyte organic matter reflect anoxic conditions. Within the YD, low oxygen conditions in Rotsee were previously reconstructed based on chitinous invertebrate remains (Ursenbacher et al. 2020). Since not all sediment cores collected in Rotsee contain *Characeae* sp. material (Lotter 1988), Characeae material is likely only preserved at local deposition centers.

Following the end of the Younger Dryas, continuous lacustrine sedimentation is observed throughout the Holocene. The absence of macrophyte layers and increase in Ca/Ti and Si/Ti values reflect a shift in the lake system (Fig. 2). Here, both Ca/Ti and Si/Ti are hypothesized to reflect an increase in phytoplankton-derived biomass, presumably supported by a watershed supply of Ca^2+^ and Si^4+^ in solution in this period. The sediments are characterized by an increasing fraction of organic carbon (Fig. 2) during the early and middle Holocene, reflecting the expected increase in trophic state with time. Substantial inputs of the watershed soils and vegetation are recognized in the macromolecular composition, with increased relative abundances of phenols and the appearance of lignin (Fig. 4). As lignin is typical for woody tissues in wood and bark, and phenols and chlorophyll are also present in plant tissues, this implies an increasing contribution of woody vegetation to the lake sediments through time. Stable contributions of carbohydrates and phenols (together ~ 50% of fractional abundance) are consistent with the notion that vascular plant-derived organic matter contributes a major portion of the sedimentary organic matter of Unit A3. However, the high contribution of vascular plant-derived organic matter in the sediments does not necessarily indicate that the original vascular plant organic matter inputs exceeded those of phytoplankton. It is also possible that the higher reactivity of phytoplankton-derived organic matter has caused a selective loss of this pool relative to more recalcitrant vascular plant-derived organic matter over time. This phenomenon of clear selective preservation of vascular plant-derived over phytoplankton-derived OM over time periods of decades and above has been shown for other lakes in the region (Han et al. 2022).

A direct effect of temperature on mass accumulation rates of inorganic and especially organic carbon is observed (Fig. 3), with an increase during the early HTM, peaking at 9 ± 0.2 cal ka BP, and during the mid-Holocene HTM. The increase in inorganic carbon MARs can either reflect increased biogenic calcite precipitating during more extensive algal blooms, or an increase in the dissolved Ca^2+^ and Si^4+^ amounts delivered to the lake in a warmer period (Gaillardet et al. 2019). An increase in organic carbon MARs is coeval with less depleted δ^15^N-TN values (Fig. 3). This can reflect an increased contribution of watershed vegetation which is generally depleted in δ^15^N (Fogel and Cifuentes 1993). Or, it represents an increase in biomass derived from nitrogen-fixing cyanobacterial blooms (δ^15^N-TN = 0.5 ‰; Fogel and Cifuentes 1993). Phytoplankton aDNA (Fig. 4) also supports the interpretation that HTM periods are characterized by a change in primary producers compared with background early Holocene conditions, with Ochrophyta (dominantly reflecting diatoms in Rotsee, in parallel with Han et al. 2022) and Chlorophyta aDNA generally absent during warm periods. The disappearance of diatoms during this proposed increase in trophic state, is however at odds with the proliferation of certain diatom species in response to man-made eutrophication in Swiss lakes (e.g. Lotter et al. 1998).

The climate warming from the Late Glacial to early Holocene thus resulted in a mixed provenance of organic matter, derived from watershed vegetation and phytoplankton-derived organic matter. Rotsee thus supports the hypothesis that climate warming that allows lake development results in increased carbon storage, with an additional impact of the Holocene Thermal Maxima.

### Sedimentary carbon storage in the Middle Holocene humic lake phase

Based on pollen and diatom assemblages presented by Lotter (1988) for Rotsee, the water depth at around 7 cal ka BP was about 5 m lower than currently, with the development of an extensive shoreline forest, an Alder carr. In addition, a relative decrease in water column pH values to 7.3 was reconstructed based on diatom diversity, a development which was coeval with the loss of the minerogenic component in Rotsee sediments (Lotter 1988). The establishment of a shallow lake system during the middle Holocene coincides with the second phase of the HTM (Fig. 3). The highest organic carbon MAR values at Rotsee, up to 180 g*m^-2^*yr^-1^, exceed the increase during the first phase of the HTM, and are thus only in part explained by increasing temperature. The inorganic carbon MAR on the other hand reaches values of zero, with no accumulation of inorganic carbon after the establishment of the Alder carr. The increased accumulation of organic carbon can be attributed firstly to a change in organic carbon provenance. The general increase through time in chlorophyll, lignin and phenol compounds throughout the early Holocene, reached a maximum value during the Alder carr phase, with the shoreline forest as a probable source (Fig. 4). The aDNA tells a similar story, while both vascular plants aDNA and lignin are present throughout the early and middle Holocene, the highest value (n=1) is observed during the Alder carr phase. The organic matter supplied to the lake system was thus derived from vegetation, rich in lignin and therefore more resistant to degradation (Martínez et al. 2005). As the large supply of vegetation-derived organic matter would have contributed to a ‘browning’ of the lake water, this could have resulted in a more shallow, warmer thermocline, and the development of an anoxic hypolimnion in Rotsee. This process has been observed in contemporary humic shallow lakes (e.g. Karpowicz and Ejsmont-Karabin 2018). While the redox-sensitive element Mn shows a relative increase during the humic lake phase, potentially indicating seasonally anoxic conditions (Fig. 3), this slightly post-dates the HTM and thus does not coincide with an increase in the organic carbon MARs. Potentially, the high organic carbon MARs occurred during a permanent anoxic stage, during which Mn was not precipitated and thus remained low in the sediments (Davison 1993; Makri et al. 2021). Following the break-up of the permanent anoxia and the release of Mn in the water column, seasonal precipitation of Mn-minerals can be the cause of the sedimentary increase in Mn/Ti ratio values.

The lack of accumulation of sedimentary TIC from 6.94 cal ka BP (± 0.19 ka) can also be explained by the development of a shallow humic lake system. The dystrophic lake chemistry associated with humic lakes, the associated brown water color and the increased amount of shading, would have impacted the amount of primary productivity and the phytoplankton composition (Jasser 1997). However, aDNA of Chlorophyta and Ochrophyta is recovered from the sediments (Fig. 4), indicating that phytoplankton remained present. A second mechanism contributing to the absence of inorganic carbonates in the shallow lake phase, could be a decreased weathering- or erosion-related input of cation-rich minerals into the lake, decreasing the alkalinity of the lake. In addition, the development of reducing and low pH conditions in the sediments can contribute to the dissolution of inorganic carbon formed in the water column. Decreased sediment pH values that result in the dissolution of TIC, can be caused by a high TOC environment (Dean 1999). Based on our data, we suggest that once the organic carbon concentration in the sediments becomes greater than 16%, the CO_2_ produced by decomposition of that OC and production of organic acids lowers the pH of anoxic pore waters enough to dissolve any inorganic carbonates that reaches the sediment–water interface (Supp. Fig. 7). However, high organic matter content is not enough to lower sedimentary pH, with sediments generally well-buffered against pH changes (Fiskal et al. 2019). A high contribution of vegetation-derived humic acids is proposed to have lowered the pH not only in the sediments, but throughout the water column. Potentially caused by an interplay of the three mechanisms—low primary productivity, low alkalinity due to reduced weathering-related input of carbonate, and dissolution due to humic acid-related low pH sediments—successional changes in lakes can thus halt inorganic carbon storage in sediments.

### Sedimentary MARs record the opening up of the Middle Holocene landscape

During the late Holocene, after approximately 6 cal ka BP, the modern atmospheric circulation pattern was established, with stronger westerly winds. The increase in precipitation and wind impact (Niessen and Kelts 1989) in Switzerland may have impacted both the nutrient input and lake level of Rotsee. Indeed, a gradual change in organic carbon composition, as reflected by a decrease in the contribution of vascular macromolecules (phenols) and aDNA between 6.5 and 4.6 cal ka BP (Fig. 4), indicates that the contribution of organic matter from the shoreline forest to the sediments was decreased. In this period, a maximum in the MARs is coeval with an increase in sedimentary Fe and P (300–260 cm blf; between 4.4 and 3.6 ± 0.2 cal ka BP). In freshwater sediments experiencing permanent anoxia, biologically produced Fe^2+^ would precipitate, either as an Fe-PO_4_ mineral, or as an FeS_x_ mineral, in the presence of HS^-^ (Rothe et al. 2016). While the sedimentary enrichment of Fe/Ti and P/Ti log-ratios hints towards the existence of this process, the absence of an increase in sedimentary S/Ti log-ratio values at the same depth, makes this interpretation less likely. Instead, we propose that the maxima in Fe and P reflect a watershed signal that has impacted the lake’s trophic level. The δ^15^N-TN value increases step-wise in the same intervals, confirming an increase in nutrients from the watershed. Although no archeological evidence of Neolithic occupation is present at Rotsee, their impact on Swiss vegetation during the Neolithic, starting at 7 ka BP, has been described (Burga 1988). Here, we propose that their transient impact on the watershed, caused an temporal increase in the Rotsee organic matter MAR. A third period of increased organic carbon MARs in Unit A2 does carry a clear signal of anthropogenic impacts. It co-occurs with increased soil erosion, as indicated by increased Ti/Al log-ratio values (Fig. 3), agreeing in time with known episodes of large forest cover removal in Switzerland during Roman times (e.g. Haas et al. 2019). This deforestation period substantially increased nutrient input from the watershed, as inferred from sedimentary δ^15^N-TN.

In Rotsee, successional changes in lake water depth, chemistry (alkalinity, pH and redox conditions), and organic matter provenance result in a large change in the amount and type of sedimentary organic matter. While the individual effects of these drivers cannot be distinguished, it is clear that millennial-scale successional changes, specifically lake infilling, potentially modulated by climate (HTM) and anthropogenic eutrophication (4 and 2 cal ka BP), promote the storage of sedimentary organic carbon.

### Human land use changes impact Late Holocene Rotsee sediments (Unit A1)

In the late Holocene, continued land use changes followed up the initial deforestation phase during Roman times, with the Ti/Al log-ratio showing two additional episodes of increased soil erosion during the Middle Ages (maxima at 644 ± 220 and 1175 to 1255 ± 210 AD), and then again in recent sediments (1949 ± 9 AD and 1961 ± 2 AD). The impact of land use changes delivering Ca-bearing minerals to the lake had a significant impact on the inorganic carbon MAR, resulting in the re-occurrence of inorganic carbon in the top 111 cm of the lake core (1120 ± 223 AD). The hypothesis that land use changes have thus fully perturbed the sedimentary carbon storage in late Holocene sediments, resulting in the re-appearance of inorganic carbon in the sediments, is thus supported.

### The impact of recent eutrophication on sedimentary carbon in the last 150 year

When comparing the current organic carbon MAR in Rotsee with Swiss lakes (Steinsberger et al. 2017), the high trophic state of the lake results in comparatively high MARs, especially in sediments of the seasonally anoxic deepest part of the lake (MAR= 172 gC*m-2*yr-1; Steinsberger et al. 2017). In the top 2–10 cm discussed here, TOC MAR varies between 100 and 115 gC *m^-2^*yr^-1^, which is lower than the reported TOC MAR in the anoxic sediments (Steinsberger et al. 2017). These offsets can be attributed to the core location, which is currently outside of the extent of the seasonally anoxic water column. At the location of the core, a peak in organic carbon MAR (150 gC*m^-2^*yr^-1^) starts at 1950 AD, corresponding with the maximum eutrophication in the 1970s (Fiskal et al. 2019). During the 1950–1970 eutrophication period, increased delivery of nutrients is confirmed by the excursion in δ^15^-TN values. During this period, the oxycline was expected to be shallower, and extended bottom water anoxia is a factor to explain the change in MAR during the last 150 years. Also the provenance and stability of the organic matter needs to be considered. The top 25 cm of the surface sediments contain a large fraction of organic matter (esters, fatty acids) that are generally not conserved over long timescales, and their decrease in fractional abundance with depth primarily reflects degradation. A small increase in phenols and lignin is coeval with the 1950–1970 eutrophication period, indicating that the increased MAR values can in part be caused by a larger contribution of watershed (soil or vegetation) derived organic matter. In contrast, labile N-containing compounds reach maximum values (17-19 %) prior to the reconstructed MAR increase, 85 to 100 years ago (12 to 28 ± 13 to 96 cal a BP; 1938 to 1922 AD), at the same time as an increase in Chlorophyta aDNA at 12 ± 13 cal a BP (1938 AD). This is interpreted as an increase in primary productivity caused by either an initial phase of anthropogenic eutrophication in Rotsee (Lotter 1989), or the trophic increase caused by the establishment of the Reuss canal in 1922. Diatom DNA only increases slightly later, from 1943 AD ± 11. The impact of anthropogenic eutrophication during the last century on lake primary productivity has thus impacted the composition of subsurface macromolecular composition, without showing a direct link with the carbon MARs. Instead, an increase in MARs was more strongly associated with an increase in soil erosion during a more limited period in time (1950 to 1970).

## Conclusion

The sedimentary carbon stored in Rotsee responds to climate-driven successional changes, from an oligotrophic macrophyte-dominated system with low carbon storage, to a phytoplankton dominated system with substantial inorganic carbon stored, to a humic shallow lake system dominated by organic sedimentary carbon. Although the amount and type of carbon changes with time, there is no long-term trend in the amount of carbon stored in Rotsee sediments over the Holocene time period. In the Late Holocene, human impacts such as deforestation or eutrophication drive carbon accumulation rates. Changes in temperature, succession, lake water chemistry and redox conditions have a compound effect on the type and amount of sedimentary carbon. The largest changes observed, from the Late Glacial to the Holocene, indicate that current climate change is most likely to impact lakes that are currently in an oligotrophic macrophyte-dominated stage. Lakes whose primary productivity is driven by phytoplankton can also show an increase in sedimentary carbon storage as a response to warming, if the parallel with the impact of the HTM in the early Holocene is made. Once human alterations in the watershed occur, soil erosional processes increase the carbon accumulation in lake sediments. As this increase goes hand in hand with a decrease of soil carbon stored in the watershed, of which part potentially gets respired during transport, it is not clear yet whether this process also results in lakes being a carbon sink. Future work could include including the carbon stocks of surrounding watershed soils, to constrain the carbon mass balance on a watershed scale.

## Supplementary Information

Below is the link to the electronic supplementary material.

Supplementary file 1 (PDF 3481 kb)

## Data Availability

All data used is made available in the Dryad data repository, at the doi 10.5061/dryad.jsxksn0mq.

## References

[CR1] Affolter S, Häuselmann A, Fleitmann D, et al (2019) Central Europe temperature constrained by speleothem fluid inclusion water isotopes over the past 14,000 years. Science Advances 5: eaav3809 10.1126/sciadv.aav3809 PMC6551184 31183398

[CR2] Ammann B (1986) Litho- and palynostratigraphy at Lobsigensee: evidences for trophic changes during the Holocene. Hydrobiologia 143:301–307

[CR3] Anderson NJ, Bennion H, Lotter AF (2014) Lake eutrophication and its implications for organic carbon sequestration in Europe. Glob Change Biol 20:2741–2751 10.1111/gcb.12584 24677531

[CR4] Bader RG (1953) (1957) The lignin fraction of marine sediments. Deep-Sea Res 4:15–22

[CR5] Baud A, Smol JP, Meyer-Jacob C et al (2023) The impacts of whole-lake acidification and eutrophication on the accumulation of lead in sediments from manipulated lakes in the Experimental Lakes Area (IISD-ELA). Environ Pollut 317:120829 36481463 10.1016/j.envpol.2022.120829

[CR6] Berg, JS, Lepine, M, Laymand, E, Han, X, Vogel, H, Morlock, MA, Gajendra, N, Gilli, A, Bernasconi, SM, Schubert, CJ, Su, G, Lever, MA, 2022. Ancient and Modern Geochemical Signatures in the 13,500-Year Sedimentary Record of Lake Cadagno. Frontiers in Earth Science 9. 10.3389/feart.2021.754888

[CR7] Bertrand S, Tjallingii R, Kylander ME, Wilhelm B, Roberts SJ, Arnaud F, Brown E, Bindler R (2024) Inorganic geochemistry of lake sediments: a review of analytical techniques and guidelines for data interpretation. Earth Sci Rev 249:104639

[CR8] Blaauw M, Christen JA (2011) Flexible paleoclimate age-depth models using an autoregressive gamma process. Bayesian Anal 6:457–474

[CR9] Burga CA (1988) Swiss vegetation history during the last 18 000 years. New Phytol 110:581–662

[CR10] Dean WE (1999) The carbon cycle and biogeochemical dynamics in lake sediments. J Paleolimnol 21:375–393

[CR11] Deng L, Bölsterli D, Kristensen E et al (2020) Macrofaunal control of microbial community structure in continental margin sediments. Proc Natl Acad Sci 117:15911–15922 32576690 10.1073/pnas.1917494117 PMC7376573

[CR12] Fiskal A, Deng L, Michel A et al (2019) Effects of eutrophication on sedimentary organic carbon cycling in five temperate lakes. Biogeosciences 16:3725–3746

[CR13] Fogel ML, Cifuentes LA (1993) Isotope fractionation during primary production. In: Engel MH, Macko SA (eds) Organic Geochemistry: Principles and Applications. Springer, US, Boston, MA, pp 73–98

[CR14] Gaillardet J, Calmels D, Romero-Mujalli G, Zakharova E, Hartmann J (2019) Global climate control on carbonate weathering intensity. Chem Geol Evol Carbonate Karst Critical Zones 527:118762

[CR15] Gajendra N, Berg JS, Vogel H et al (2023) Carbohydrate compositional trends throughout Holocene sediments of an alpine lake (Lake Cadagno). Front Earth Sci 11:1047224

[CR16] Gudasz C, Bastviken D, Steger K et al (2010) Temperature-controlled organic carbon mineralization in lake sediments. Nature 466:478–481 20651689 10.1038/nature09186

[CR17] Haas M, Baumann F, Castella D et al (2019) Roman-driven cultural eutrophication of Lake Murten, Switzerland. Earth Planet Sci Lett 505:110–117

[CR18] Han X, Tolu J, Deng L, et al (2022) Long-term preservation of biomolecules in lake sediments: potential importance of physical shielding by recalcitrant cell walls. PNAS Nexus 1:pgac076 10.1093/pnasnexus/pgac076 PMC9896894 36741427

[CR19] Hasler AD (1947) Eutrophication of lakes by domestic drainage. Ecology 28:383–395

[CR20] Heiri O, Lotter AF (2003) 9000 years of chironomid assemblage dynamics in an Alpine lake: long-term trends, sensitivity to disturbance, and resilience of the fauna. J Paleolimnol 30:273–289

[CR21] Hofmann A, Roussy D, Filella M (2002) Dissolved silica budget in the North basin of Lake Lugano. Chem Geol 182:35–55

[CR22] Höhn L, Leunda M, Gobet E et al (2022) Vegetation response to rapid climate change during the Lateglacial-Early Holocene transition at Gola di Lago, southern Switzerland. Boreas 51:606–620

[CR23] Jasser I (1997) The dynamics and importance of picoplankton in shallow, dystrophic lake in comparison with surface waters of two deep lakes with contrasting trophic status. Hydrobiologia 342:87–93

[CR24] Karpowicz M, Ejsmont-Karabin J (2018) Influence of environmental factors on vertical distribution of zooplankton communities in humic lakes. Ann Limnol Int J Limnol 54:17

[CR25] Kastowski M, Hinderer M, Vecsei A (2011) Long-term carbon burial in European lakes: Analysis and estimate. Global Biogeochemical Cycles 25 (3): GB3019

[CR26] Lazzaretti MA, Hanselmann KW, Brandl H et al (1992) The role of sediments in the phosphorus cycle in Lake Lugano. II. Seasonal and spatial variability of microbiological processes at the sediment-water interface. Aquatic Sci 54:285–299

[CR27] Lever MA, Torti A, Eickenbusch P et al (2015) A modular method for the extraction of DNA and RNA, and the separation of DNA pools from diverse environmental sample types. Front Microbiol 6:476 26042110 10.3389/fmicb.2015.00476 PMC4436928

[CR28] Lim J, Lee J-Y, Hong S-S et al (2019) Holocene coastal environmental change and ENSO-driven hydroclimatic variability in East Asia. Quatern Sci Rev 220:75–86

[CR29] Liu X, Colman SM, Brown ET et al (2013) Estimation of carbonate, total organic carbon, and biogenic silica content by FTIR and XRF techniques in lacustrine sediments. J Paleolimnol 50:387–398

[CR30] Lotter AF (1989) Subfossil and modern diatom plankton and the paleolimnology of Rotsee (Switzerland) since 1850. Aquatic Sci 51:338–350

[CR31] Lotter AF (1998) The recent eutrophication of Baldeggersee (Switzerland) as assessed by fossil diatom assemblages. The Holocene 8:395–405

[CR32] Lotter AF, Birks HJB, Eicher U et al (2000) Younger Dryas and Allerød summer temperatures at Gerzensee (Switzerland) inferred from fossil pollen and cladoceran assemblages. Palaeogeogr Palaeoclimatol Palaeoecol 159:349–361

[CR33] Lotter AF (1988) Paläoökologische und paläolimnologische Studie des Rotsees bei Luzern. Dissertationes Botanicae, Band 124. Publisher: Schweizerbart Science Publishers, Germany.

[CR34] Martínez AT, Speranza M, Ruiz-Dueñas FJ et al (2005) Biodegradation of lignocellulosics: microbial, chemical, and enzymatic aspects of the fungal attack of lignin. Int Microbiol 8:195–204 16200498

[CR35] Meyers S (2014) astrochron: A Computational Tool for Astrochronology. 1.4

[CR36] Moscariello A, Schneider AM, Filippi ML (1998) Late glacial and early Holocene palaeoenvironmental changes in Geneva Bay (Lake Geneva, Switzerland). Palaeogeogr Palaeoclimatol Palaeoecol 140:51–73

[CR37] Moser KA, Baron JS, Brahney J et al (2019) Mountain lakes: eyes on global environmental change. Global Planet Change 178:77–95

[CR38] Müller B, Meyer JS, Gächter R (2016) Alkalinity regulation in calcium carbonate-buffered lakes. Limnol Oceanogr 61:341–352

[CR39] Nicolussi K, Kaufmann M, Patzelt G et al (2005) Holocene tree-line variability in the Kauner Valley, Central Eastern Alps, indicated by dendrochronological analysis of living trees and subfossil logs. Veget Hist Archaeobot 14:221–234

[CR40] Niessen F, Kelts K (1989) The deglaciation and Holocene sedimentary evolution of southern perialpine Lake Lugano: implications for Alpine paleoclimate. Eclogae Geol Helv 82:235–263

[CR41] Ninnes S, Tolu J, Meyer-Jacob C et al (2017) Investigating molecular changes in organic matter composition in two Holocene lake-sediment records from central Sweden using pyrolysis-GC/MS. JGR Biogeosciences 122:1423–1438

[CR42] Norris MW, Turnbull JC, Howarth JD, Vandergoes MJ (2020) Pretreatment of terrestrial macrofossils. Radiocarbon 62:349–360

[CR43] Olsen J, Björck S, Leng MJ et al (2010) Lacustrine evidence of Holocene environmental change from three Faroese lakes: a multiproxy XRF and stable isotope study. Quatern Sci Rev 29:2764–2780

[CR44] Pastorino P, Elia AC, Pizzul E et al (2024) The old and the new on threats to high-mountain lakes in the Alps: a comprehensive examination with future research directions. Ecol Ind 160:111812

[CR45] Reber R, Akçar N, Ivy-Ochs S et al (2014) Timing of retreat of the Reuss Glacier (Switzerland) at the end of the Last Glacial Maximum. Swiss J Geosci 107:293–307

[CR46] Reimer PJ, Austin WEN, Bard E et al (2020) The IntCal20 Northern Hemisphere Radiocarbon Age Calibration Curve (0–55 cal kBP). Radiocarbon 62:725–757

[CR47] Renssen H, Seppä H, Crosta X et al (2012) Global characterization of the Holocene Thermal Maximum. Quatern Sci Rev 48:7–19

[CR48] Rothe M, Kleeberg A, Hupfer M (2016) The occurrence, identification and environmental relevance of vivianite in waterlogged soils and aquatic sediments. Earth Sci Rev 158:51–64

[CR49] Samartin S, Heiri O, Vescovi E, et al (2012) Lateglacial and early Holocene summer temperatures in the southern Swiss Alps reconstructed using fossil chironomids. Journal of Quaternary Science 27:279–289

[CR50] Stadelmann P (1980) Der Zustand des Rotsees bei Luzern, in: Maihof-Rotsee. Geschichte und Eigenart eines Quartiers, edited by: Quartierverein Maihof, Quartierverein Maihof, Luzern, 54–61,25 (in German)

[CR51] Steinsberger T, Schmid M, Wüest A et al (2017) Organic carbon mass accumulation rate regulates the flux of reduced substances from the sediments of deep lakes. Biogeosciences 14:3275–3285

[CR52] Teranes JL, Bernasconi SM (2000) The record of nitrate utilization and productivity limitation provided by δ^15^N values in lake organic matter—a study of sediment trap and core sediments from Baldeggersee, Switzerland. Limnol Oceanogr 45:801–813

[CR53] Tolu J, Gerber L, Boily J-F, Bindler R (2015) High-throughput characterization of sediment organic matter by pyrolysis–gas chromatography/mass spectrometry and multivariate curve resolution: a promising analytical tool in (paleo)limnology. Anal Chim Acta 880:93–102 26092342 10.1016/j.aca.2015.03.043

[CR54] Trautmann S, Knoflach B, Stötter J et al (2023) Potential impacts of a changing cryosphere on soils of the European Alps: a review. CATENA 232:107439

[CR55] Ursenbacher S, Stötter T, Heiri O (2020) Chitinous aquatic invertebrate assemblages in Quaternary lake sediments as indicators of past deepwater oxygen concentration. Quatern Sci Rev 231:106203

[CR56] Verbruggen F, Heiri O, Reichart G-J, Lotter AF (2010) Chironomid δ^18^O as a proxy for past lake water δ^18^O: a Lateglacial record from Rotsee (Switzerland). Quatern Sci Rev 29:2271–2279

[CR57] Vollweiler N, Scholz D, Mühlinghaus C et al (2006) A precisely dated climate record for the last 9 kyr from three high alpine stalagmites, Spannagel Cave. Austria Geophys Res Lett 33(20):L20703

[CR58] Welten M (1982) Vegetationsgeschichtliche Untersuchung in den westlichen schweizer Alpen. Published by Birkhauser, Boston (USA), Bern - Wallis

[CR59] Wick L, Tinner W (1997) Vegetation changes and timberline fluctuations in the central alps as indicators of holocene climatic oscillations. Arct Alp Res 29:445–458

[CR60] Willerslev E, Hansen AJ, Binladen J et al (2003) Diverse plant and animal genetic records from holocene and pleistocene sediments. Science 300:791–795 12702808 10.1126/science.1084114

[CR61] Zhao Y, Liu Z, Colin C et al (2011) Turbidite deposition in the southern South China Sea during the last glacial: evidence from grain-size and major elements records. Chin Sci Bull 56:3558–3565

[CR62] Züllig H, Rheineck S (1985) Pigmente phototropher Bakterien in Seesedimenten und ihre Bedeutung für die Seenforschung. Schweiz Z Hydrol 47:87–126

