# Supplementary material for: Holocene environmental change in Rotsee and its impact on sedimentary carbon storage
Source: J Paleolimnol. 2025 Jun 11;73(4):311–27. doi: 10.1007/s10933-025-00361-3 (PMC12241216; doi:10.1007/s10933-025-00361-3)
Supplement: Supplementary file 1 — (PDF 3481 kb) [file 10933_2025_361_MOESM1_ESM.pdf]

Supplementary Materials to the manuscript:

Holocene environmental change in Rotsee and its impact on sedimentary carbon storage

Cindy De Jonge[1], Nathalie Dubois[2], S. Nemiah Ladd[3], Longhui Deng[4], Niroshan Gajendra[4, 5], Negar Haghipour[1, 6], Carsten J. Schubert[4, 7], Mark Lever[4, 8]

[1] Geological Institute, ETH Zurich, Sonneggstrasse 5, 8092 Zurich, Switzerland

[2] Department of Surface Waters Research and Management, Eawag, Uberlandstrasse 133, 8600 Dubendorf, Switzerland.

[3] Department of Environmental Sciences, University of Basel, Bernoullistrasse 30/32, 4056 Basel, Switzerland

[4] Institute of Biogeochemistry and Pollutant Dynamics, ETH Zurich, Universitaetstrasse 16, Zurich, Switzerland

[5] Currently at: Institute for Energy Technology (IFE), Instituttveien 18, 2007, Kjeller, Norway

[6] Laboratory of Ion Beam Physics, ETH Zurich, Switzerland.

[7] Department of Surface Waters, EAWAG, Swiss Federal Institute of Aquatic Science and Technology, Kastanienbaum, Switzerland.

[8] Currently at: Marine Science Institute, University of Texas at Austin, 750 Channel View Drive, Port Aransas, TX 78373, USA.

(A) labID	Type	210Pb(Bq/kg)	2*sd(210Pb)	composite depth (cm blf)	thickness(cm)	226Ra(Bq/kg)	2*sd(226Ra)
ROT21-9C_01	210Pb	150	19.1	1	1	0.027	3.97
ROT21-9C_02	210Pb	122.6	16.9	2	1	0.0244	4.68
ROT21-9C_03	210Pb	120.1	15.2	3	1	0.0219	4.27
ROT21-9C_04	210Pb	126.4	18.2	4	1	0.0226	4.98
ROT21-9C_05	210Pb	145.1	17	5	1	0.0229	3.59
ROT21-9C_06	210Pb	143.8	20.3	6	1	0.0232	4.94
ROT21-9C_07	210Pb	134.5	17.6	7	1	0.0215	4.2
ROT21-9C_08	210Pb	132.2	16.6	8	1	0.0238	3.87
ROT21-9C_09	210Pb	124.2	16.5	9	1	0.0258	4.07
ROT21-9C_11	210Pb	76.4	11.5	11	1	0.021	5
ROT21-9C_13	210Pb	75.1	11	13	1	0.0208	4.32
ROT21-9C_14	210Pb	72.4	9.8	14	1	0.0251	3.74
ROT21-9C_15	210Pb	64.4	9.4	15	1	0.026	3.91
ROT21-9C_16	210Pb	53.8	5.5	16	1	0.0236	2.57
ROT21-9C_17	210Pb	48.5	8	17	1	0.0284	4.77
ROT21-9C_19	210Pb	21.7	3.9	19	1	0.0217	3.85
ROT21-9C_20	210Pb	34.9	5.4	20	1	0.0249	4.49
ROT21-9C_21	210Pb	28.3	5.5	21	1	0.0221	5.18
ROT21-9C_22	210Pb	24.5	4.3	22	1	0.0227	4.73
ROT21-9C_23	210Pb	26.6	3.7	23	1	0.0238	4.18
ROT21-9C_25	210Pb	19.3	3.7	25	1	0.0218	4.09
ROT21-9C_27	210Pb	18.6	3.8	27	1	0.0226	5.02
ROT21-9C_29	210Pb	20.8	4.2	29	1	0.0218	5.01
ROT21-9C_31	210Pb	6.9	1.7	31	1	0.0237	4.06
ROT21-9C_33	210Pb	8.1	2.1	33	1	0.0194	5.29
ROT21-9C_35	210Pb	8.9	2.4	35	1	0.0199	4.7
ROT21-9C_37	210Pb	2.8	0.5	37	1	0.0232	3.46
ROT21-9C_38	210Pb	8.4	1.7	38	1	0.0244	3.59
ROT21-9C_39	210Pb	4.3	0.9	39	1	0.0249	3.55
ROT21-9C_40	210Pb	2.4	0.5	40	1	0.024	3.44
ROT21-9C_42	210Pb	10.5	2.2	42	1	0.024	4.71
ROT21-9C_45	210Pb	12	2	45	1	0.0238	3.29
ROT21-9C_48	210Pb	2.9	0.7	48	1	0.0229	3.48
ROT21-9C_50	210Pb	10.6	2	50	1	0.023	3.5

(B) labID	Type	age	error (yr)	composite depth (cm blf)
Cs_1	137Cs peak	35	1	14.5
Cs_2	137Cs peak	58	1	19.5
ROT21_1_A_45	14C, leaf fragment	77	68	55.3
ROT21_1_B_66	14C, leaf fragment	2008	72	176.8
ROT21_1_C_30.5	14C, leaf fragment	3656	81	241.3
ROT21_1_C_55	14C, conifer needle	3353	74	265.8
ROT21_1_C_56.5	14C, leaf fragment	3866	184	267.3
ROT21_1_C_80	14C, beech nut	3436	75	290.8
ROT21_2_B_45	14C, leaf fragment, fragment of beech nut	5233	81	430.8
ROT21_2_C_64	14C, leaf and twig fragments	6806	88	549.8
ROT21_3_B_15	14C, seedpods of grass	8016	92	673.8
ROT21_3_B_84	14C, leaf fragment	10047	98	742.8
ROT21_4_B_3.5	14C, leaf fragments	8860	103	838.8
ROT21_4_C_5	14C, fibrous material	8840	108	940.8
ROT21_4_C_65.5	14C, Chara stem *	9680	268	984.8
ROT21_4_C_70	14C, Chara stem *	10296	252	989.3
ROT21_4_C_76	14C, Chara stem *	10229	257	995.3
ROT21_4_C_81	14C, Chara stem *	11005	264	1000.3
ROT21_4_C_87	14C, Chara stem *	11425	262	1006.3
ROT21_4_C_90.5	14C, Chara stem *	11311	290	1009.8
ROT21_4_C_92	14C, Chara stem *	10926	259	1011.3

(C) labID	Reservoir derived 14C age	Reservoir derived 14C error	Atmosphere derived 14C age	Atmosphere derived 14C error	Reservoir age offset	Reservoir age offset error
Shallow (1-B-66, 1-B-63)	2686	71	2008	72	678	101
Deep (4-C-5, 4-C-14)	10169	98	8840	108	1329	146

Supp. Table 1. The ages and associated offsets of the ^{210}Pb, ^{137}Cs and ^{14}C dates used for the age model (Fig. 1A). The estimated reservoir ages of section 4C (C) are used to correct for the ^{14}C ages of the macrophyte macrofossil remains.

Compound class	Compound	Compound class	Compound
Alcohol	1-Dodecanol, 2-hexyl- (C18:0)		1H-Pyrrole-2,5-dione
	Behenic alcohol		Benzyl nitrile
	Ethanol, 2-(octadecyloxy)-		3-Pyridinol, 6-methyl-
Aldehyde	Tetradecanal		Benzenepropanenitrile
Alkane	Heneicosane (C22:0)		Indole
Alkene	6-Tridecene (C13:1)		Diketodipyrrole
	1-Docosene (C22:1)		cis-Cyclo(L-Ala-L-Pro)
Carbohydrate	Furfural		Cyclo(Pro-Gly)
	Furan, 2,3-dihydro-2,5-dimethyl-		Cyclo(Pro-Pro)
	2-Furancarboxaldehyde, 5-methyl-		Alkylamide2
	2-Furancarboxaldehyde, 5-methyl-		Cyclo(Pro-Lys-NH3)
	2-Cyclohexen-1-one, 4-hydroxy-	PAHs	p-Xylene
	4-hydroxy-5,6-dihydro(2H)-pyran-2-one		Ethanone, 2,2-dihydroxy-1-phenyl-
	D-Limonene		1H-Indole, 3-methyl-
	Dianhydrorhamnose		2H-1-Benzopyran-3,4-diol, 2-(3,4dimethoxyphenyl)-3,4-dihydro-6-methyl-, (2α,3α,4α)-
	Benzofuran, 2,3-dihydro-		Butan-2-one, 4-(3-hydroxy-2-methoxyphenyl)-
	3-Acetamidofuran	Phenol	Phenol
	Methyl-α-d-ribofuranoside		p-Cresol
	1,6-Anhydro-β-d-talopyranose		Phenol, 2-methoxy-
	Levoglucosan		Phenol, 3-ethyl-
	Methyl N-acetyl-d-glucosamide		Creosol
Ester	Decanoic acid, decyl ester		Phenol, 4-ethyl-2-methoxy-
	Dodecanoic acid, tetradecyl ester		2-Methoxy-4-vinylphenol
	Decanoic acid, decyl ester		Phenol, 2,6-dimethoxy-
	Dodecanoic acid, tetradecyl ester		Phenol, 2-methoxy-5-(1-propenyl)-, (E)-
	Dodecanoic acid, hexadecyl ester		Phenol, 2-methoxy-4-(1-propenyl)-
	Dodecanoic acid, tetradecyl ester		Phenol, 2,6-dimethoxy-4-(2-propenyl)-
	Tetradecanoic acid, hexadecyl ester		4-((1E)-3-Hydroxy-1-propenyl)-2-methoxyphenol
Fatty Acid	2-Pentenoic acid (C5:1)	Chlorophyll	Prist-1-ene
	n-Hexadecanoic acid (C16:0)	Sterane	Cholesta-3,5-diene
	Octadecanoic acid (C18:0)		Stigmastan-3,5-diene
	2-Heptadecanone (C17:1)	Other	Friedelan-3-one
Hopene	22,29,30 trisnorhop17(21)-ene		γ-Tocopherol
	Urs-20-en-16-one		
Ketone	2-Heptadecanone (C17:1)		
	2-Pentadecanone		
	2-Nonadecanone		
Lignin	Syringaldehyde		
N-compound	1H-Pyrrole, 3-methyl-		
	dl-α-Methylglutamic acid		
	4(3H)-Pyrimidinone, 3-methyl-		

Supp. Table 2. The molecular composition (compound diversity) of the Py-GC/MS compound classes.

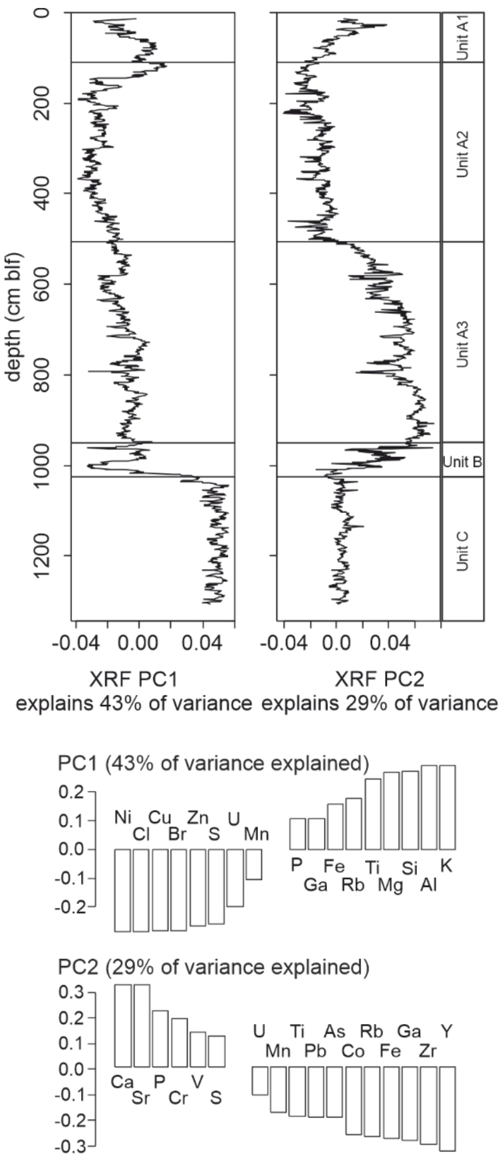

Supp. Fig. 1. The downcore scores of the principal components of variation (PC1, PC2), based on a principal component analysis of the scaled clr-transformed XRF counts, with the loading of the individual elements on PC1 and PC2 represented by barplots, using a cut-off value (score>0.1).

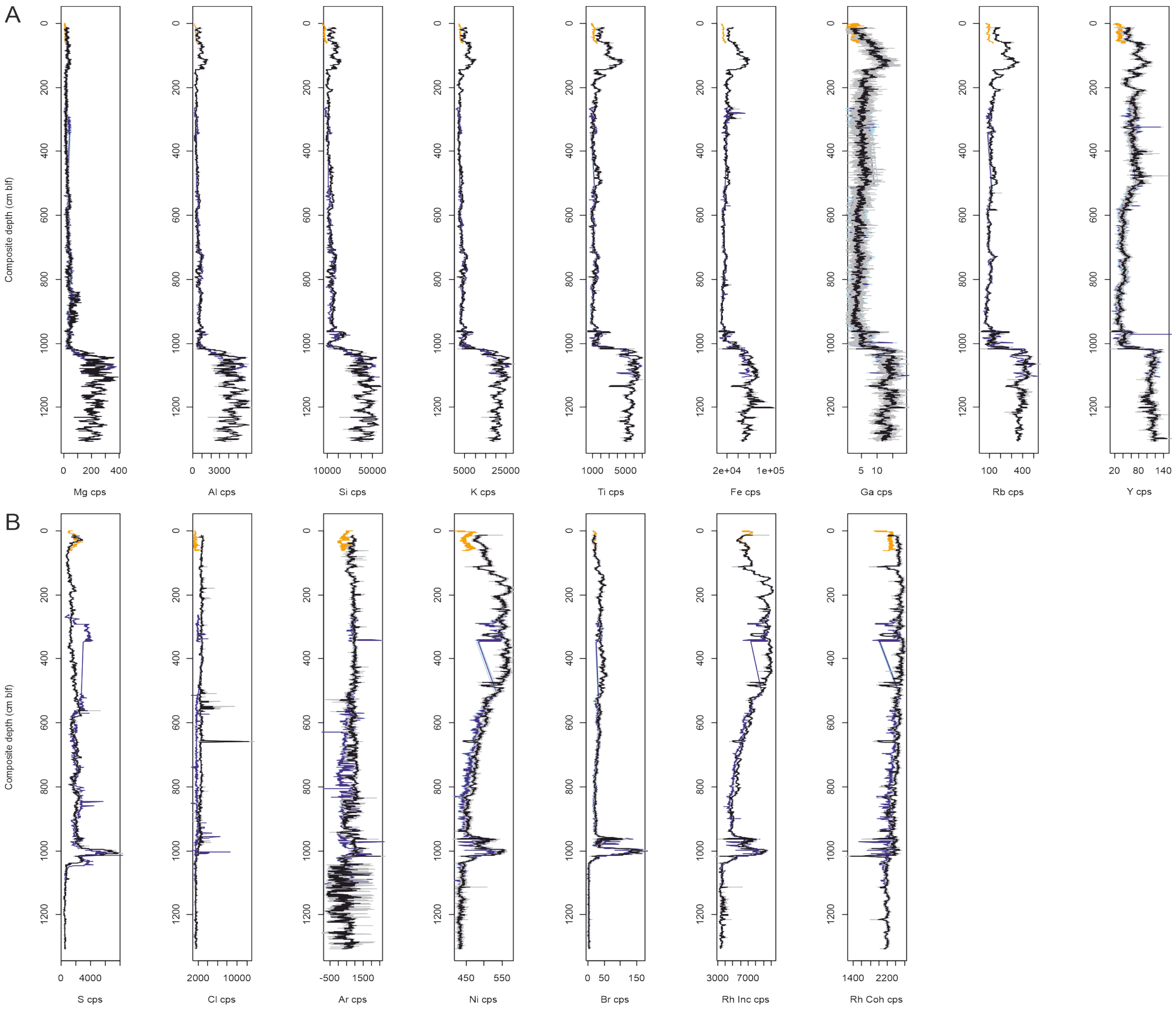

A
B
Composite depth (cm blf)
0
200
400
600
800
1000
1200
Mg cps
0 200 400
Al cps
0 3000
Si cps
10000 50000
K cps
5000 25000
Ti cps
1000 5000
Fe cps
2e+04 1e+05
Ga cps
5 10
Rb cps
100 400
Y cps
20 80 140
S cps
0 4000
Cl cps
2000 10000
Ar cps
-500 1500
Ni cps
450 550
Br cps
0 50 150
Rh Inc cps
3000 7000
Rh Coh cps
1400 2200

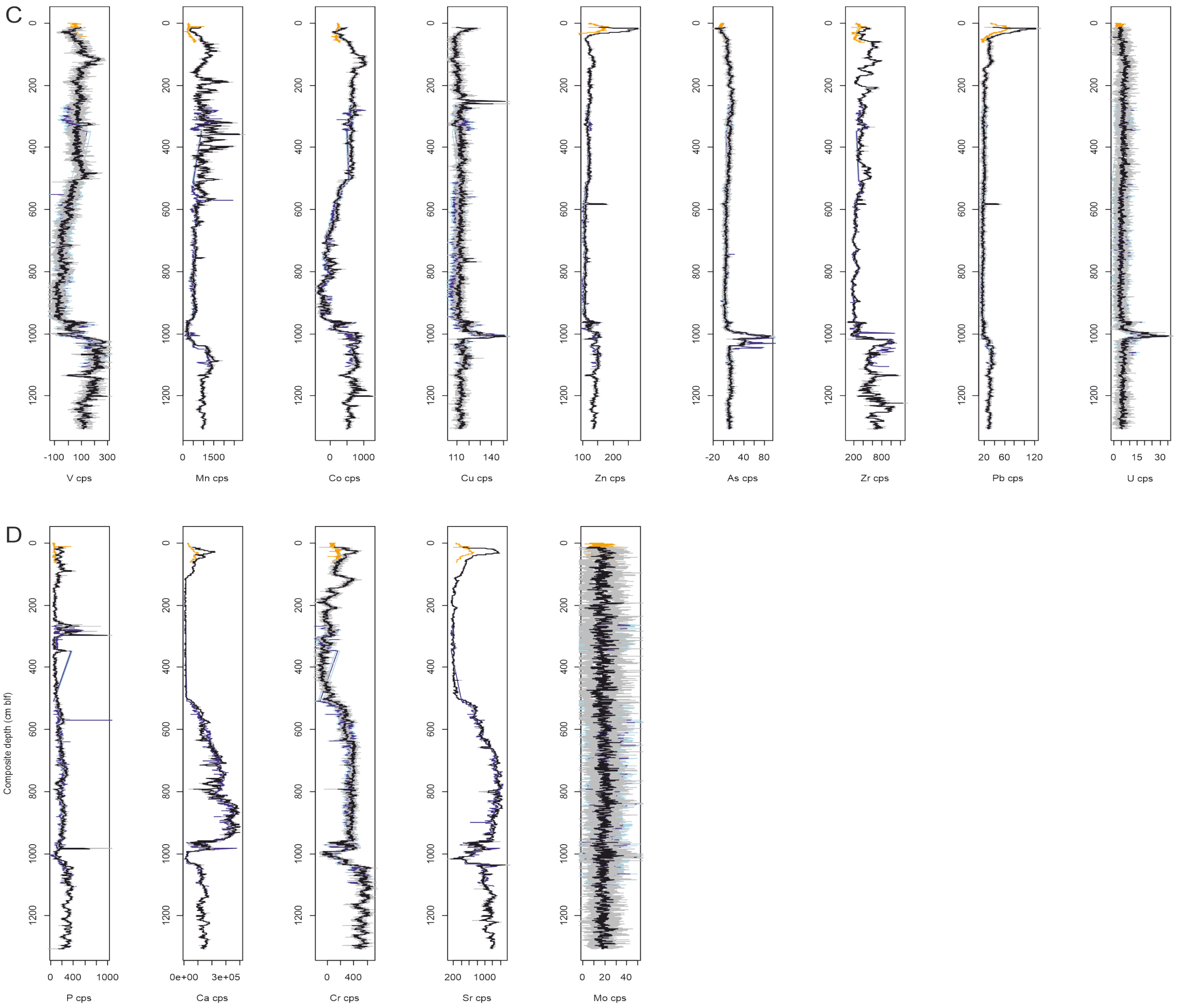

Supp. Fig. 2. The XRF counts of all analysed elements, plotted as a 2-point average. Black indicates XRF measurements done on the first borehole, blue indicates measurements done on the second borehole. Grey indicates the standard deviation of the measurements.

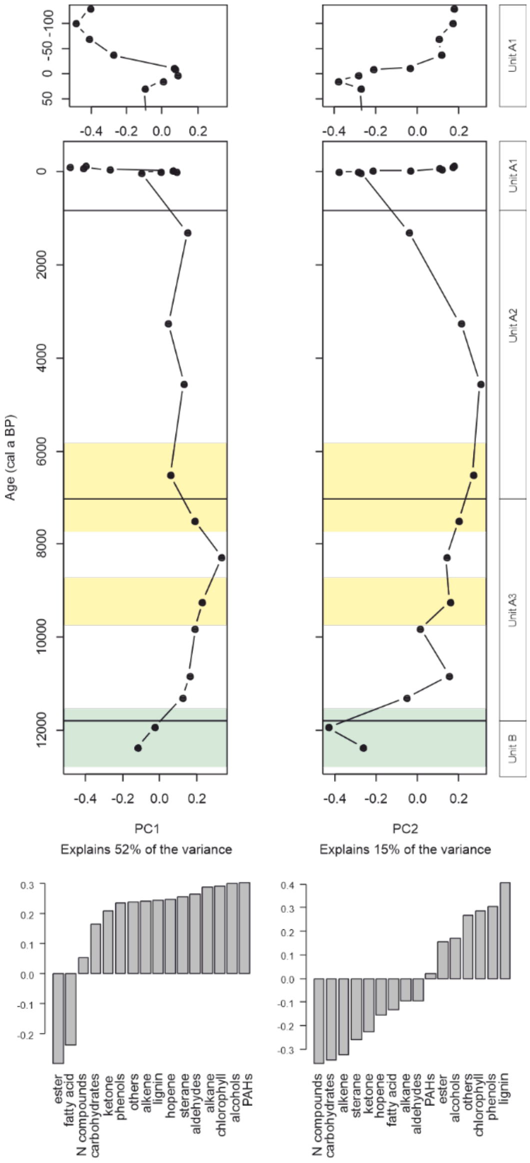

Supp. Fig. 3. The downcore scores of the principal components of variation (PC1, PC2) based on the standardized relative abundances of the compound classes (Fig. 4), with the loading of the individual compound classes on PC1 and PC2 represented by barplots. Background colors indicate climate periods, with Younger Dryas indicated in green and Holocene warm periods indicated in yellow. The most recent 100 years are replotted on a more detailed scale.

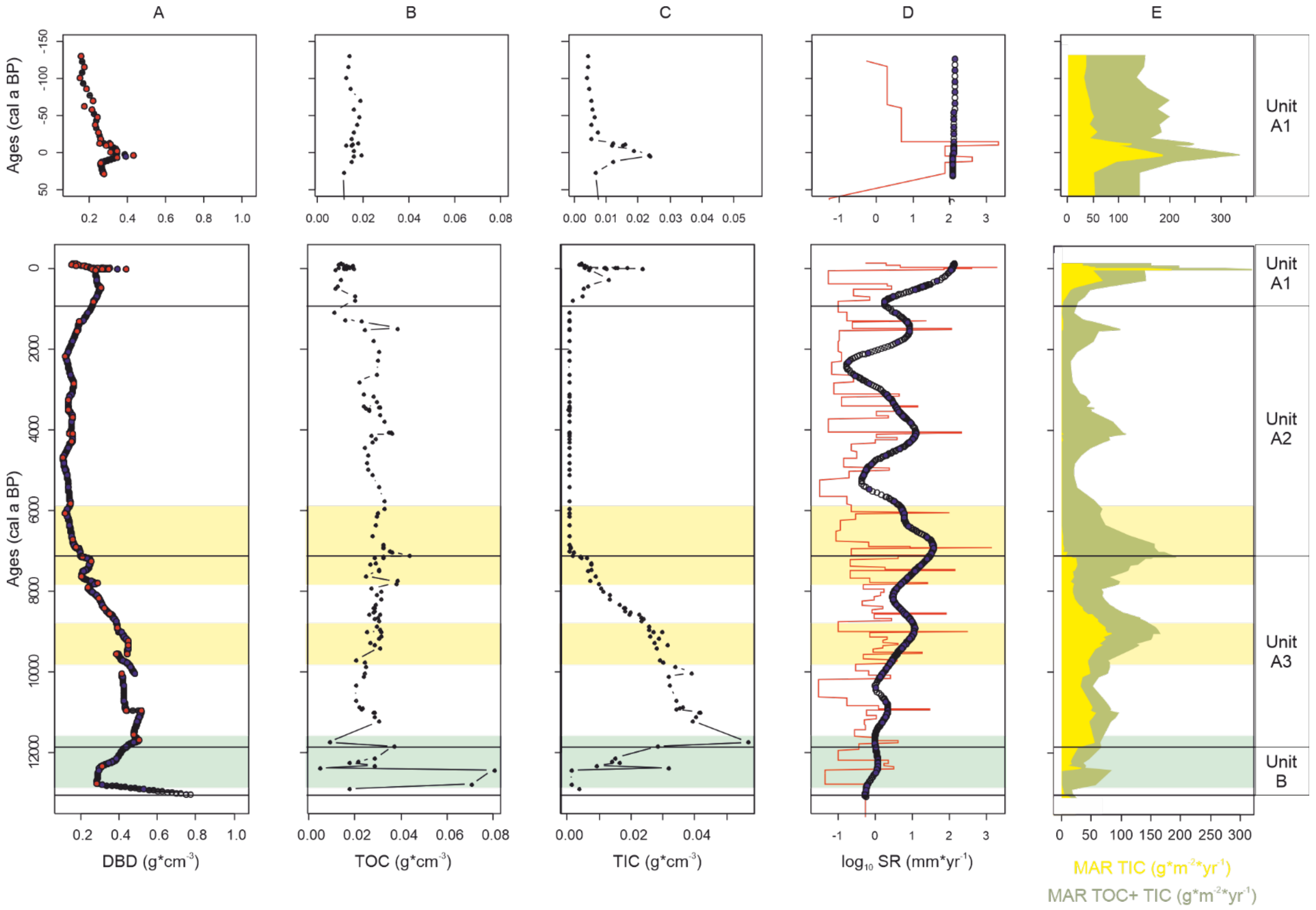

Supp. Fig. 4. The downcore values of the A) measured (red symbol) and interpolated (black and blue symbols, with blue symbols indicating depths selected for downstream calculations) dry bulk density values (g cm^{-3}), B) calculated total organic carbon and C) total inorganic carbon density (g cm^{-3}), D) the sedimentation rate (red line), with smoothed trend (black and blue symbols, with blue symbols indicating depths selected for downstream calculations). These parameters (B-D) are at the basis of the calculated MAR rates for total inorganic carbon (TIC) [yellow area] and summed TIC and total organic carbon (TOC) [green area], plotted in panel E. Background colors indicate climate periods, with Younger Dryas indicated in green and Holocene warm periods indicated in yellow. For all parameters, the most recent 100 years are replotted on a more detailed scale.

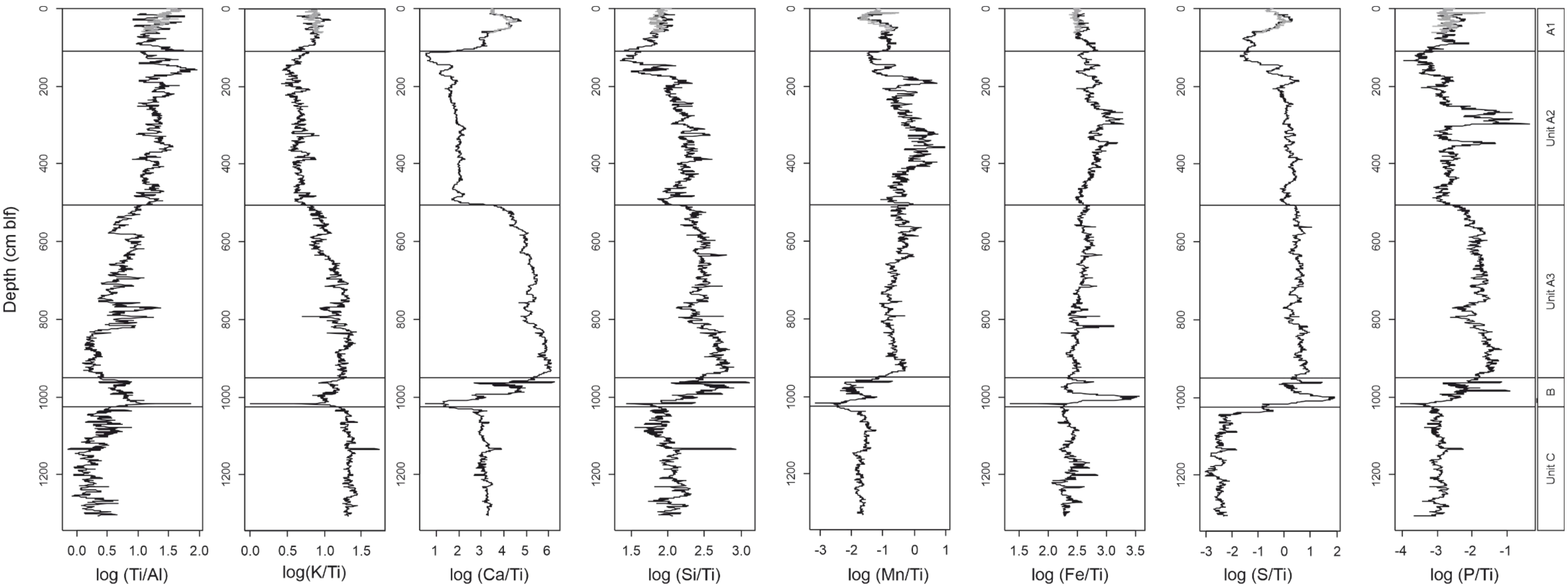

Supp. Fig. 5. Selected XRF log-ratios, plotted against depth. The grey data points reflect the XRF ratio values measured on the short core.

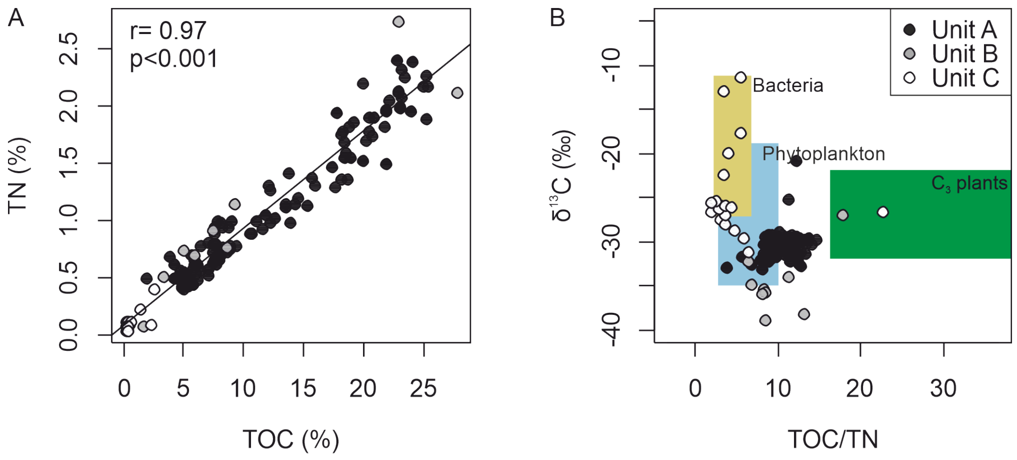

Supp. Fig. 6. Bulk organic matter parameters, A) scatterplot of total organic carbon (TOC, %) and total nitrogen (TN, %) and B) $\delta^{13}C_{\text{-TOC}}$ and C/N plot after Meyers et al. (1997). Colored areas indicate organic matter provenance brackets after Lamb et al. (2006). Symbol colors reflect the lithological Units A-C (see legend).

Meyers PA (1997) Organic geochemical proxies of paleoceanographic, paleolimnologic, and paleoclimatic processes. Organic Geochemistry 27:213–250. https://doi.org/10.1016/S0146-6380(97)00049-1

Lamb AL, Wilson GP, Leng MJ (2006) A review of coastal palaeoclimate and relative sea-level reconstructions using $\delta^{13}C$ and C/N ratios in organic material. Earth-Science Reviews 75:29–57. https://doi.org/10.1016/j.earscirev.2005.10.00

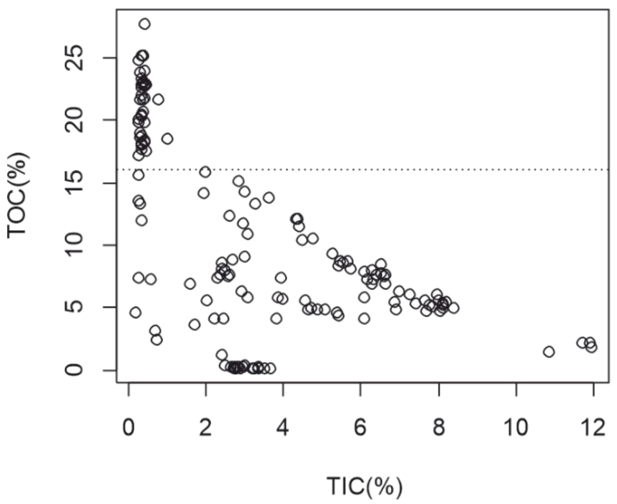

Supp. Fig. 7. A scatterplot showing the dependency between total inorganic carbon (TIC) and total organic carbon (TOC) content of the lacustrine sediments. The horizontal line indicates the cut-off value of 16% TOC.
